# Elevated serum homocysteine levels associated with poor recurrence-free and overall survival in patients with colorectal cancer

**DOI:** 10.1038/s41598-024-60855-4

**Published:** 2024-05-02

**Authors:** Hailun Xie, Lishuang Wei, Qiwen Wang, Shuangyi Tang, Jialiang Gan

**Affiliations:** 1 Department of Gastrointestinal Gland Surgery, The First Affiliated Hospital, Guangxi Medical University, 6 Shuangyong Road, Nanning, 530021 Guangxi People’s Republic of China; 2 Department of Geriatric Respiratory Disease Ward, The First Affiliated Hospital, Guangxi Medical University, Nanning, Guangxi People’s Republic of China; 3 Department of Pharmacy, The First Affiliated Hospital, Guangxi Medical University, Nanning, Guangxi People’s Republic of China; 4 Department of Colorectal and Anal Surgery, The First Affiliated Hospital of Guangxi Medical University, 6 Shuangyong Road, Nanning, 530021 Guangxi People’s Republic of China; 5 Guangxi Key Laboratory of Enhanced Recovery After Surgery for Gastrointestinal Cancer, Nanning, 530021 Guangxi People’s Republic of China

**Keywords:** Malnutrition, Homocysteine, Nomogram, Colorectal cancer, Prognostic, Cancer, Gastrointestinal diseases

## Abstract

This study aimed to evaluate the significance of homocysteine (HCY) levels in predicting recurrence-free survival (RFS) and overall survival (OS) in colorectal cancer (CRC) patients. This retrospective study involved 1272 CRC patients. The risk of mortality increased with increasing HCY levels in CRC patients. The optimal HCY cutoff value in CRC patients was 15.2 μmol/L. The RFS (45.8% vs. 60.5%, p < 0.001) and OS (48.2% vs. 63.2%, p < 0.001) of patients with high HCY levels were significantly lower than those of patients with low HCY levels. Patients with high HCY levels were older, male, had large tumours, high carcinoembryonic antigen (CEA) levels, and long hospital stays, and incurred high hospitalisation costs. Multivariate analysis showed that when HCY levels exceeded 15.2 μmol/L, the risk of adverse RFS and OS increased by 55.7% and 61.4%, respectively. Subgroup analysis showed that HCY levels could supplement CEA levels and pathological staging. We constructed HCY-based prognostic nomograms, which demonstrated feasible discrimination and calibration values better than the traditional tumour, node, metastasis staging system for predicting RFS and OS. Elevated serum HCY levels were strongly associated with poor RFS and OS in CRC patients. HCY-based prognostic models are effective tools for a comprehensive evaluation of prognosis.

## Introduction

Colorectal cancer (CRC) is a common malignancy worldwide. According to the latest cancer statistics, CRC ranks third in terms of incidence and second in terms of mortality among all malignancies^1^. In recent years, the incidence and mortality of CRC in China have continued to increase, with CRC ranking second in terms of incidence and fourth in terms of mortality among all malignancies^2–4^. Surgical treatment remains the primary therapeutic approach for CRC patients. The prognosis of CRC is mainly influenced by the completeness of surgical resection and pathological staging. Although patients who are diagnosed at an early stage of CRC can achieve a 5-year survival rate of over 90% through surgical treatment, a majority of patients are diagnosed at advanced stages, leading to a poor prognosis^2,5–7^.

Chronic inflammation is a major risk factor that affects intestinal health and forms the basis for the occurrence and development of CRC^8–10^. Systemic or localised metabolic disorders, such as nutrient deficiencies, may exacerbate the inflammatory response within the intestine, thereby increasing the risk of adverse prognosis in CRC patients. Deficiency of vitamin B 9, folate, is a risk factor for colorectal cancer while homocysteine (HCY), an important intermediate metabolite in one-carbon metabolism, is gaining increasing attention owing to its sensitivity to functional folate deficiency^11–13^. Increased levels of HCY may lead to abnormal deoxyribonucleic acid (DNA) methylation. Abnormal methylation is considered an important factor that promotes tumour invasion, lymph node metastasis, and distant metastasis^14,15^. Moreover, HCY can serve as an indicator of chronic inflammation, and its elevated levels may contribute to the occurrence or progression of colorectal cancer through inflammatory mechanisms. High levels of HCY are highly prevalent in patients with inflammatory bowel disease^16,17^. HCY is an indicator of obesity, hyperinsulinaemia, and chronic inflammation and is strongly associated with colorectal cancer. Martínez et al. found that markers related to folate metabolism, including elevated HCY levels, are significantly associated with an increased recurrence of colorectal adenomas^18^. Bobe et al. discovered that serum HCY levels could be an effective indicator of dietary inflammation, which is closely linked to the recurrence of colorectal adenomas^19^. In summary, HCY serves as a comprehensive reflection of metabolic disturbances and chronic inflammation and holds great potential for widespread application in predicting the prognosis of CRC patients.

However, there is currently limited research on the relationship between HCY levels and the prognosis of CRC patients. Therefore, we conducted a single-centre retrospective cohort study to explore the value of HCY levels in predicting recurrence-free survival (RFS) and overall survival (OS) in CRC patients. Our goal was to individually monitor the recurrence risk and survival outcomes of colorectal cancer patients by monitoring HCY levels and constructing a prediction model based on HCY levels, thus providing more guidance and references for clinical work.

## Materials and methods

### Population

The current study included patients who underwent surgical treatment at the First Affiliated Hospital of Guangxi Medical University between 2015 and 2017. The following patients were included: (1) primary cancer located in the colon or rectum, undergoing curative surgery, and with a postoperative pathological diagnosis confirming colorectal cancer; (2) complete serum laboratory test data for five days before the surgery; (3) complete postoperative follow-up data; and (4) age ≥ 18 years. The exclusion criteria were patients who received neoadjuvant therapy before surgery, patients with concurrent acute or chronic inflammatory diseases (acute upper respiratory tract infection, pneumonia, acute pancreatitis, acute appendicitis, and pyelonephritis), patients with confirmed severe liver/kidney disease before surgery (hepatitis, cirrhosis, end-stage renal disease), and patients with concomitant malignant tumours, and patients who engage in habitual smoking and excessive alcohol consumption.

### Data collection

To gather comprehensive information, we used the hospital information system to collect admission records. These records included basic personal information such as patient sex, age, height, weight, presence of hypertension and diabetes, and other relevant details. Additionally, we conducted preoperative blood sampling and performed laboratory tests on the blood samples. During this process, we examined complete blood counts and tumour markers such as carcinoembryonic antigen (CEA). We also obtained information on postoperative radiotherapy and chemotherapy by reviewing the patients’ medical records. Finally, from the pathological results, we acquired detailed data on tumour, node, metastasis (TNM) staging (based on the 8th edition of the American Joint Committee on Cancer staging), perineural/vascular invasion, and tumour size and differentiation. Normal CEA was defined as < 5 ng/mL, while high CEA was defined as ≥ 5 ng/mL.

### Follow-up methods

The main follow-up method used in this study was telephone consultation, followed by regular outpatient visits. Typically, patients were followed up at 3–6 months intervals during the first year after surgery, and then once a year thereafter until the patient's death. The follow-up content mainly included the postoperative recovery of patients, regular check-ups, occurrence of recurrence or metastasis, timing of recurrence or metastasis, and time and cause of death. The last follow-up date recorded in this study was January 2023. The average follow-up duration was 61.0 months (0.3 months–106.8 months).

OS was calculated from the date of hospital admission to the patient's death for any reason, measured in months. RFS was calculated from the date of hospital admission to the occurrence of either recurrence or death, whichever came first (measured in months).

### Statistical analysis

To compare categorical variables, we conducted analyses using the chi-square test. For the analysis of continuous variables, we used Student's t-test. To determine the optimal cutoff value of HCY levels for the prognosis of CRC patients, we employed maximally selected rank statistics. We also utilised restricted cubic splines (RCS) fitted to Cox proportional hazards models and set three nodes at the 25th, 50th, and 75th percentiles to flexibly model the relationship between HCY levels and RFS/OS of CRC patients. To evaluate the association between HCY levels and survival, we constructed survival curves using the Kaplan–Meier method and compared the survival rates using the log-rank test. Univariate and multivariate Cox proportional hazard regression analyses were used to assess the independent risk factors for RFS/OS in CRC patients and the corresponding hazard ratios (HRs) and 95% confidence intervals (CIs). We evaluated the relationship between HCY levels and the RFS/OS of CRC patients by treating HCY as a continuous variable, a binary variable, and a four-category variable (with HCY divided into four equal parts). For feature selection, using the least absolute shrinkage and selection operator (LASSO) logistic regression algorithm and including the selected meaningful variables in the multivariate Cox regression model. Using significant variables from the multivariate Cox regression model, we constructed prognostic nomograms and assessed their prognostic accuracy using measures, such as the concordance index (C-index), calibration curve, and receiver operating characteristic (ROC) curve. In the calibration plots, we divided the patients into four equal groups, with each blue dot representing a group of patients. The x-axis represents the predicted probabilities by the model, while the y-axis represents the actual probability. The line connecting the dots represents the calibration curve, which illustrates the relationship between the predicted probabilities and the actual observations. Finally, we employed decision curve analysis (DCA) to compare the clinical utility of the models. A significance level of p < 0.05 was considered statistically significant throughout all the analyses. All statistical analyses were using R software (version 4.0.2).

### Ethics committee approval

This study followed the Helsinki declaration. All participants signed an informed consent form and this study was approved by the ethics committee of the First Affiliated Hospital, Guangxi Medical University (Registration number: NO.2022-KY-(043)).

## Results

### Baseline clinicopathological characteristics

The current study included 1272 patients, of which 806 were male (63.4%) and 466 were female (36.6%). The mean age was 59.2 years (± 12.65). 623 patients had colon cancer (49.0%) and 649 had rectal cancer (51.0%). According to the clinical and pathological diagnoses, 679 patients (46.6%) had stages I–II disease and 593 patients (46.6%) had stages III–IV disease. The HCY levels of patients ranged from 1.13 to 55.00, with a mean of 12.99 and a median of 12.45. The maximally selected rank statistics method was used to determine the optimal cut-off value of HCY levels for CRC patients, which was found to be 15.2 μmol/L (Supplementary Fig. S1). A total of 942 patients were classified into the low HCY level group (< 15.2 μmol/L) and 330 patients were classified into the high HCY level group (≥ 15.2 μmol/L).

High HCY levels were significantly associated with male sex, old age, presence of hypertension, large tumour diameter, and high CEA levels. Furthermore, compared to the low HCY level group, the high HCY level group had an overall increased mortality rate of 15.0% (36.8% vs. 51.8%, p < 0.001) and an increased recurrence rate of 6.3% (25.8% vs. 32.1%, p = 0.032). Patients in the high HCY level group had a prolonged hospital stay of two days and an increased hospital cost of 2232.92 RMB (Supplementary Table S1). Further exploration of the median HCY level distribution in various clinical and pathological features revealed that male sex, age ≥ 60 years, high body mass index (BMI), and CRC patients who died were factors for higher HCY levels (Supplementary Fig. S2).

### Kaplan–Meier survival analysis of low and high HCY levels

**Figure 1:**
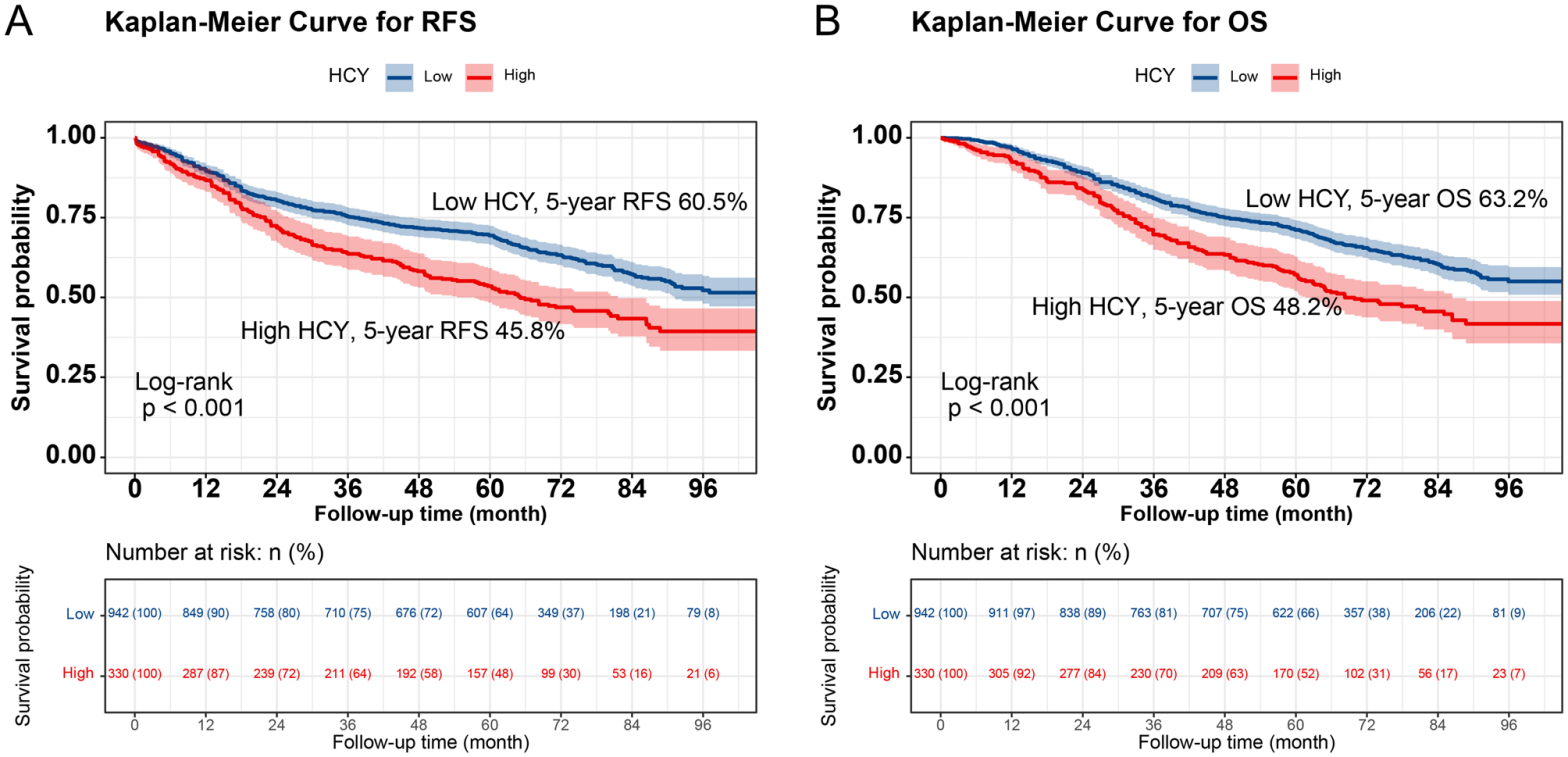
Kaplan–Meier curve of HCY levels in patients with colorectal cancer. (**A**) Kaplan–Meier curve for RFS; (**B**) Kaplan–Meier curve for OS. *RFS* recurrence-free survival, *OS* overall survival.

**Figure 2:**
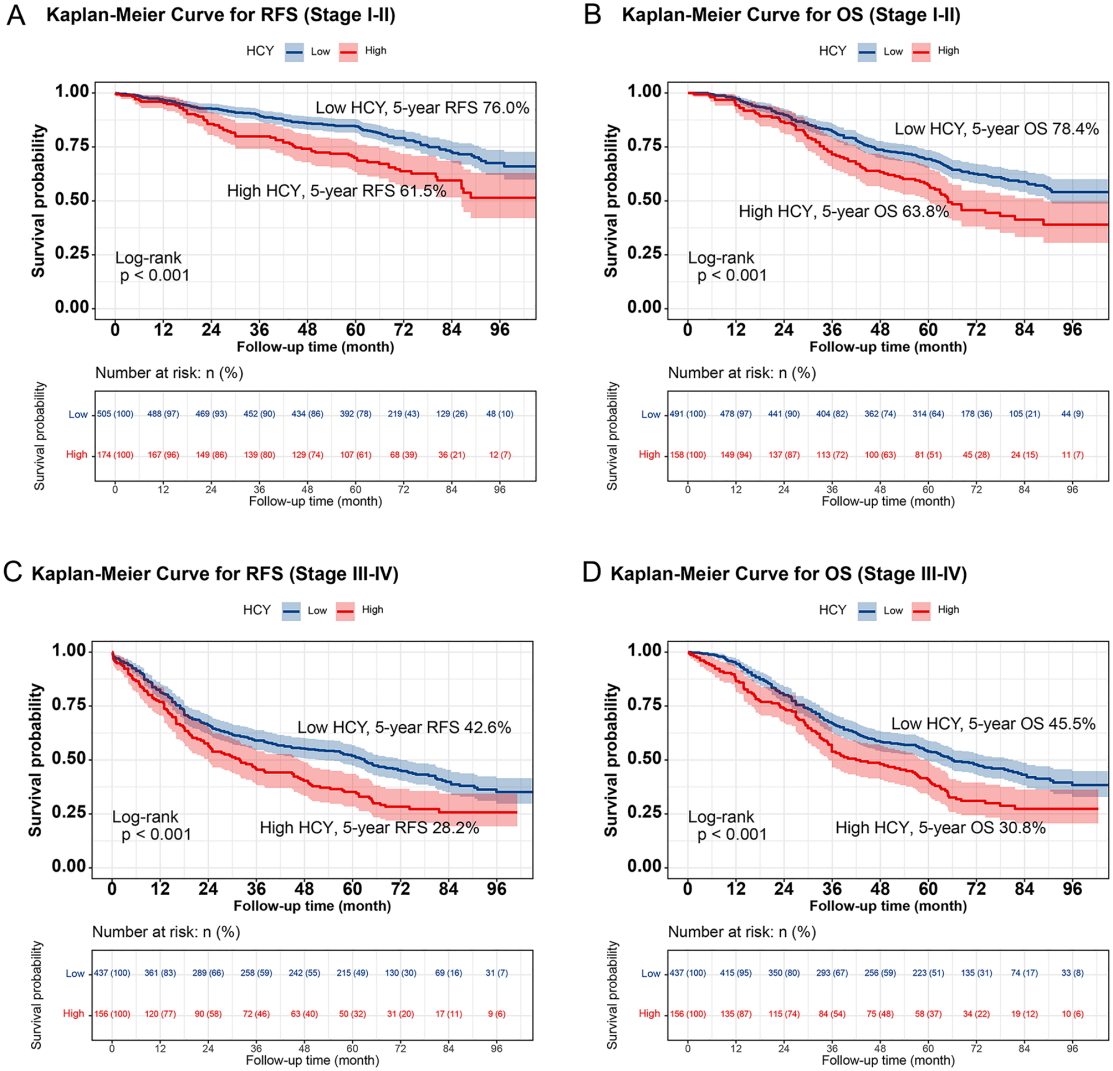
Stratified Kaplan–Meier curve of HCY levels based on TNM stage subgroup in patients with colorectal cancer. (**A**) RFS (Stage I–II); (**B**) OS (Stage I–II); (**C**) RFS (Stage III–IV); (**D**) RFS (Stage III–IV). *RFS* recurrence-free survival, *OS* overall survival.

In total, 551 patients (43.3%) experienced the RFS outcome, which includes both recurrence and death. Kaplan–Meier survival curves showed that the 5-year RFS rate of CRC patients with high HCY levels was significantly lower than that of CRC patients with low HCY levels (45.8% vs. 60.5%, p < 0.001) (Fig. 1A). A subgroup analysis based on TNM staging revealed that HCY levels significantly differentiated the RFS of patients with stage I–II CRC (61.5% vs. 74.6%, p < 0.001) and those with stage III–IV CRC (28.2% vs. 42.6%, p < 0.001) (Fig. 2A,C). The OS in high HCY patients was observably lower than that in low HCY patients (48.2% vs. 63.2%; p < 0.001) (Fig. 1B). For stages I–II, HCY levels effectively differentiated the OS of CRC patients (63.8% vs. 78.4%, p < 0.001) (Fig. 2B). Similar patterns were observed for stages III–IV (30.8% vs. 45.5%, p < 0.001) (Fig. 2D).

Then, we explored the prognostic value of HCY levels in different tumour types. For colon cancer, patients in the high HCY level group had significantly lower RFS (47.1% vs. 63.9%, p < 0.001) and OS (50.0% vs. 65.4%, p < 0.001) than those in the low HCY level group (Supplementary Fig. S3A,B). For rectal cancer, patients in the high HCY level group had lower RFS (44.3% vs. 57.4%, p < 0.001) and OS (46.2% vs. 61.1%, p < 0.001) than those in the low HCY level group (Supplementary Fig. S4A,B). Subsequently, we explored the value of CEA levels. In the normal CEA level group, patients in the high HCY level group had a lower RFS (57.8% vs. 67.3%, p = 0.014) and OS (61.3% vs. 69.8%, p = 0.024) than those in the low HCY level group (Supplementary Fig. S5A,B). In the high CEA level group, HCY levels could still effectively differentiate the RFS (32.5% vs. 50.7%, p < 0.001) and OS (33.8% vs. 53.5%, p < 0.001) of CRC patients and had a better differentiating effect than in the normal CEA level group (Supplementary Fig. S6A,B). Subsequently, we conducted the analysis of the combined effect of CEA and HCY. We defined the group with low CEA and low HCY as CEA-HCY I, the group with low CEA and high HCY as CEA-HCY II, the group with high CEA and low HCY as CEA-HCY III, and the group with high CEA and high HCY as CEA-HCY IV. We found that the combination of CEA and HCY could significantly further distinguish the PFS and OS of CRC patients (Supplementary Figs. S6B, S7A). Multivariate Cox proportional hazard regression analyses revealed that the combination of CEA and HCY was an independent prognostic factor for CRC patients. Compared to the CEA-HCY I group, the HR for prognosis in the CEA-HCY II, CEA-HCY III, and CEA-HCY IV groups were 1.596, 1.496, and 2.203, respectively (Supplementary Table S2).

### Prognostic values of HCY levels in CRC patients

**Figure 3:**
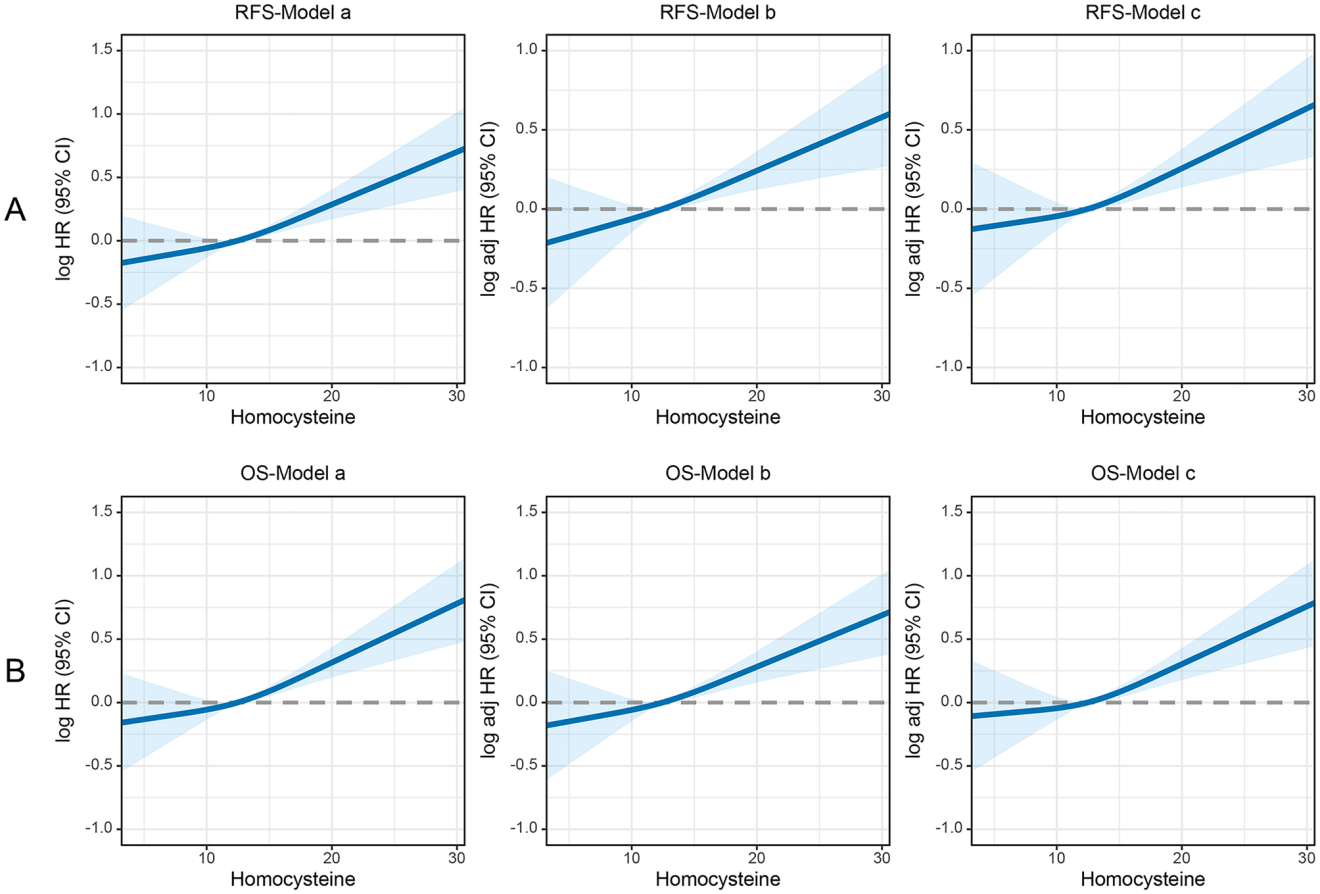
Restricted cubic splines to flexibly model and visualize the relation of predicted HCY with mortality in patients with colorectal cancer. (**A**) Restricted cubic splines for RFS; (**B**) Restricted cubic splines for OS. Model a: No adjusted. Model b: Adjusted for age, sex, BMI, T stage, N stage, M stage. Model c: Adjusted for sex, age, BMI, T stage, N stage, M stage, tumor location, tumor size, perineural invasion, vascular invasion, differentiation, radiotherapy, chemotherapy, hypertension, diabetes, family history. *RFS* recurrence-free survival, *OS* overall survival.

As shown in Fig. 3A, RCS were used to flexibly model and visualise the association between HCY levels and RFS. The results showed a continuous increase in the risk of adverse RFS as HCY levels increased. The RCS plot showed an inverted L-shaped relationship between HCY levels and OS, indicating a continuous increase in the risk of death with increasing HCY levels (Fig. 3B). Cox regression analysis of RFS revealed that for each SD increase in HCY levels, the risk of adverse RFS in CRC patients increased by 16.4% (HR 1.164, 95% CI 1.075–1.261, p < 0.001). Compared with patients in the low HCY level group (< 15.2 μmol/L), those in the high HCY level group (≥ 15.2 μmol/L) had a 55.7% increased risk of adverse RFS (HR 1.557, 95% CI 1.290–1.879, p < 0.001). HCY levels were divided into quartiles, with the lowest quantile (Q1) used as the reference. Q2 (9.90–12.45), Q3 (12.45–15.30), Q4 (11.12–16.48), and Q5 (≥ 15.30) were all associated with adverse outcome (p < 0.001). Adjusting for confounding factors, the HRs for RFS were 0.80, 1.027, and 1.425, respectively (Table 1).

**Table 1:** Association between HCY and RFS of patients with colorectal cancer.

IBI	Model a	p value	Model b	p value	Model c	p value
Continuous (per SD)	1.188 (1.103,1.28)	< 0.001	1.158 (1.071,1.253)	< 0.001	1.164 (1.075,1.261)	< 0.001
Cutoff value (High)	1.566 (1.31,1.872)	< 0.001	1.512 (1.254,1.823)	< 0.001	1.557 (1.29,1.879)	< 0.001
Quartiles						
Q1 (~ 9.90)	Ref		Ref		Ref	
Q2 (9.90 ~ 12.45)	0.899 (0.699,1.155)	0.405	0.829 (0.638,1.078)	0.161	0.802 (0.615,1.047)	0.104
Q3 (12.45 ~ 15.30)	1.089 (0.854,1.389)	0.49	1.084 (0.831,1.414)	0.552	1.027 (0.783,1.346)	0.848
Q4 (15.30 ~)	1.563 (1.243,1.966)	< 0.001	1.432 (1.106,1.854)	0.006	1.425 (1.097,1.851)	0.008
p for trend		< 0.001		< 0.001		< 0.001

Model a: No adjusted.

Model b: Adjusted for age, sex, BMI, T stage, N stage, M stage.

Model c: Adjusted for sex, age, BMI, T stage, N stage, M stage, tumor location, tumor size, perineural invasion, vascular invasion, differentiation, radiotherapy, chemotherapy, hypertension, diabetes, family history.

Multivariate Cox analysis of OS also showed that HCY level was an independent prognostic factor for CRC patients (HR 1.199, 95% CI 1.103–1.302, p < 0.001). Patients in the high HCY level group (≥ 15.2 μmol/L) also had a higher risk of adverse OS than those in the low HCY level group (< 15.2 μmol/L), with a 61.4% increased risk (HR 1.614, 95% CI 1.330–1.960, p < 0.001). Compared with patients with low HCY levels (Q1), those in the high HCY level group (Q3 and Q4) had a higher risk of adverse OS, with HRs of 0.805, 1.041, and 1.486, respectively (Table 2). We conducted multivariate subgroup. The results showed that high HCY levels were an independent risk factor affecting RFS (Supplementary Fig. S7) and OS (Supplementary Fig. S8) in most subgroups of CRC patients.

**Table 2:** Association between HCY and OS of patients with colorectal cancer.

IBI	Model a	p value	Model b	p value	Model c	p value
Continuous (per SD)	1.21 (1.121,1.307)	< 0.001	1.188 (1.095,1.288)	< 0.001	1.199 (1.103,1.302)	< 0.001
Cutoff value (high)	1.611 (1.341,1.935)	< 0.001	1.584 (1.307,1.919)	< 0.001	1.614 (1.33,1.960)	< 0.001
Quartiles						
Q1 (~ 9.90)	Ref		Ref		Ref	
Q2 (9.90 ~ 12.45)	0.892 (0.687,1.158)	0.39	0.816 (0.622,1.071)	0.143	0.805 (0.611,1.061)	0.124
Q3 (12.45 ~ 15.30)	1.099 (0.855,1.412)	0.461	1.084 (0.824,1.426)	0.564	1.041 (0.787,1.377)	0.778
Q4 (15.30 ~)	1.611 (1.271,2.041)	< 0.001	1.493 (1.143,1.95)	0.003	1.486 (1.134,1.948)	0.004
p for trend		< 0.001		< 0.001		< 0.001

Model a: No adjusted.

Model b: Adjusted for age, sex, BMI, T stage, N stage, M stage.

Model c: Adjusted for sex, age, BMI, T stage, N stage, M stage, tumor location, tumor size, perineural invasion, vascular invasion, differentiation, radiotherapy, chemotherapy, hypertension, diabetes, family history.

### OS and RFS prediction models

**Figure 4:**
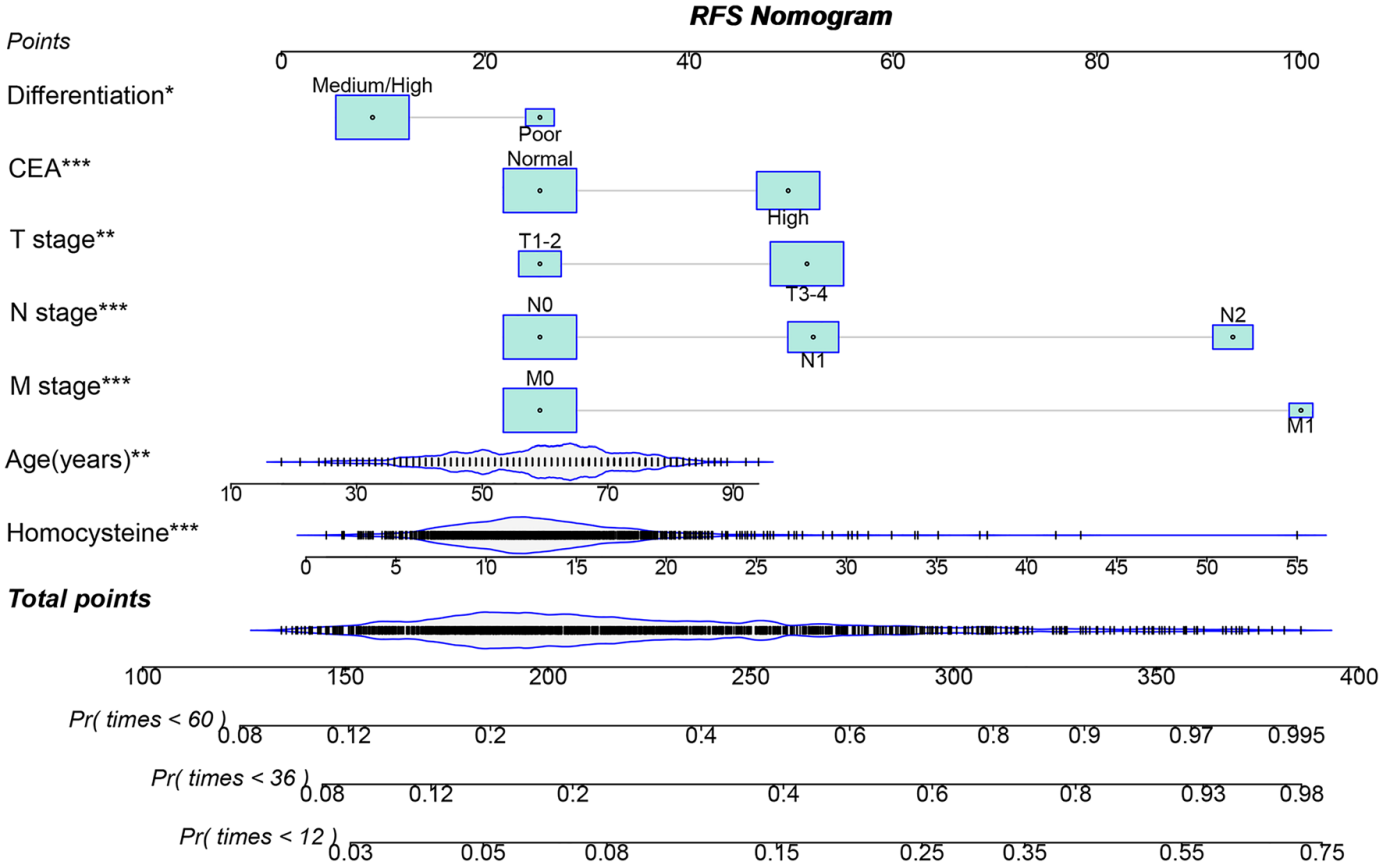
Construction the HCY-based RFS nomograms in CRC patients. Notes: The nomogram is composed of specific clinical features, with each feature corresponding to a specific point. The score for each feature can be calculated by drawing a straight line along the point axis, and the sum of these feature scores is then positioned on the total point axis. The risk probability can be calculated by drawing downward to the predicted axis. *RFS* recurrence-free survival, *CRC* colorectal cancer. Normal CEA was defined as < 5 ng/mL, while high CEA was defined as ≥ 5 ng/mL.

**Figure 5:**
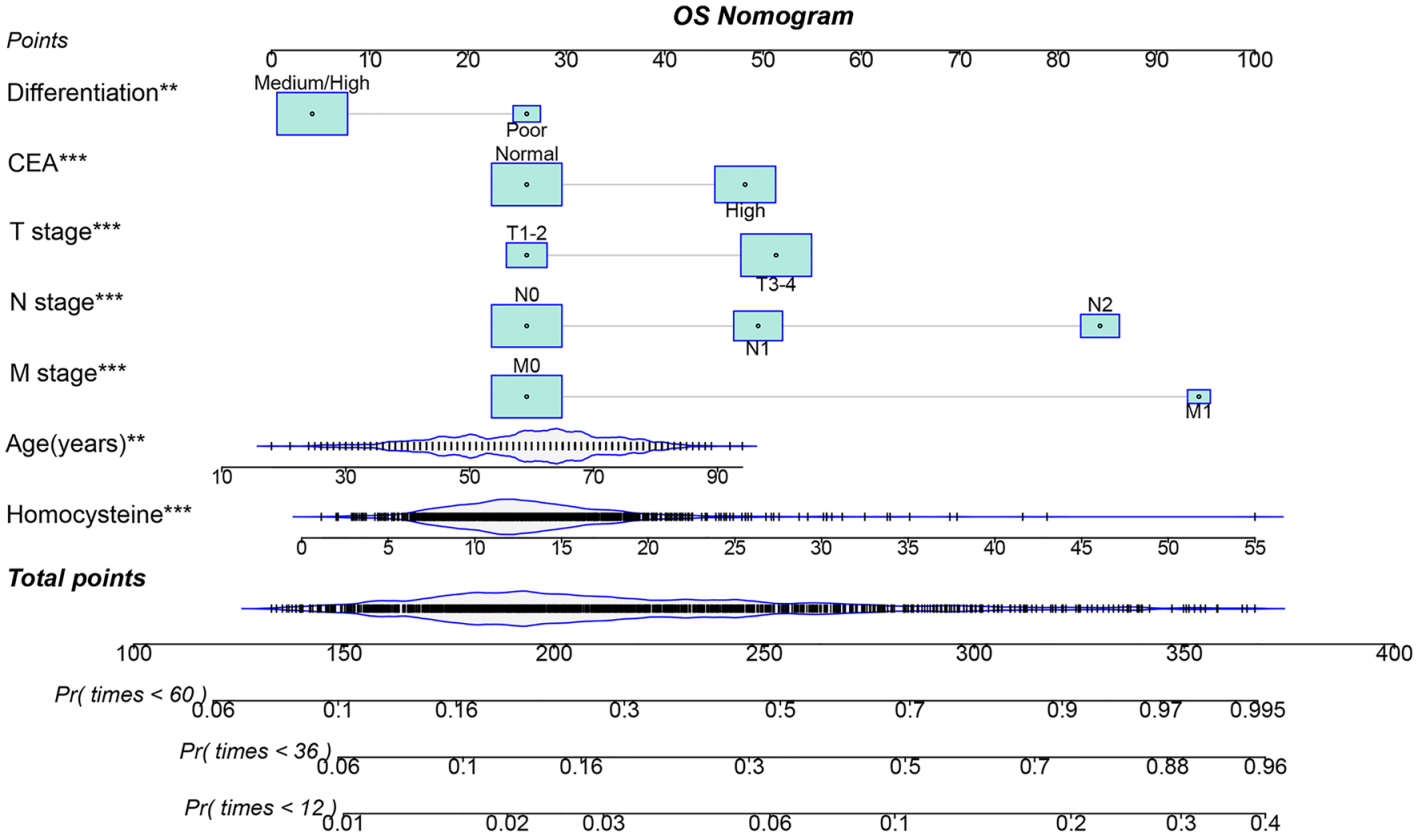
Construction the HCY-based OS nomograms in CRC patients. The nomogram is composed of specific clinical features, with each feature corresponding to a specific point. The score for each feature can be calculated by drawing a straight line along the point axis, and the sum of these feature scores is then positioned on the total point axis. The risk probability can be calculated by drawing downward to the predicted axis. *OS* overall survival, *CRC* colorectal cancer. Normal CEA was defined as < 5 ng/mL, while high CEA was defined as ≥ 5 ng/mL.

We used the LASSO logistic regression algorithm to select the most effective prognostic features for CRC patients. When the optimal lambda values for RFS and OS were 0.003 and 0.007, respectively (Supplementary Fig. S9), we identified 18 and 16 nonzero coefficient features as the best prognostic features for RFS and OS, respectively. Subsequently, we incorporated these features into a multivariate Cox regression model (Supplementary Tables S3 and S4). We determined that seven features were independent factors influencing the RFS and OS of patients, differentiation, age, T, N, and M stages, serum CEA levels, and HCY levels. Furthermore, based on these seven features, we created prognostic nomograms for predicting RFS and OS (Figs. 4 and 5). From these nomograms, we can observe that as CEA levels increase, poor differentiation occurs, the TNM stage progresses, and age, HCY levels, and predictive scores increase, indicating an increased risk of adverse prognosis.

The C-indices of the RFS and OS nomograms were 0.721 (95% CI 0.699–0.743) and 0.729 (95% CI 0.706–0.752), respectively. The Brier score for the RFS nomogram was 0.157, while the OS nomogram had a Brier score of 0.139. These scores being less than 0.25 indicate that the predictive results were effective. We used ROC curves to evaluate the accuracy of the nomograms in predicting the prognosis of CRC patients. The 1-year, 3-year, and 5-year area under the ROC curves (AUCs) of the RFS nomograms were 0.796, 0.779, and 0.768, respectively (Supplementary Fig. S10A). Similarly, the AUCs of the OS nomogram at 1-year, 3-year, and 5-year follow-ups were 0.763, 0.777, and 0.770, respectively (Supplementary Fig. S10B). Next, we assessed the calibration of the nomogram using calibration plots. The results showed good consistency between the predicted and observed values for the 1-year, 3-year, and 5-year RFS and OS nomograms (Supplementary Fig. S11A,B). Furthermore, we evaluated the clinical utility of our constructed prognostic nomogram compared with the traditional TNM staging system using DCA. We found that the RFS nomogram was superior to the TNM staging system in predicting the 1-year, 3-year, and 5-year RFS in CRC patients (Supplementary Fig. S12A). Similarly, for predicting the 1-year, 3-year, and 5-year OS, the OS nomogram model was better than the TNM staging system (Supplementary Fig. S12B). We divided the patients into high- and low-scoring groups based on median scores from the nomograms. Patients with high scores had significantly worse RFS and OS compared to those with low scores (all p < 0.001) (Supplementary Fig. S13A,B).

## Discussion

HCY is not an encoded amino acid, but it can undergo metabolic conversion towards HCY-thiolactone catalyzed by methionyl-tRNA synthetase (MARS). Subsequently, HCY-thiolactone reacts chemically with protein lysine residues, forming lysine homocysteinylation (K-Hcy) proteins. Due to its disruption of protein structure/function, K-Hcy modification is associated with several diseases^20,21^. Wang et al.^22^ also reported that high-fat diet-induced organ-specific colon K-Hcy elevation may promote CRC pathogenesis by impeding DNA damage repair. Although many studies have confirmed the significant association between high serum HCY levels and the occurrence of CRC, there is insufficient evidence regarding the adverse long-term prognosis of high HCY levels in CRC patients who undergo surgical treatment^18,19,23^.

In the current study, we confirmed that high HCY level is an independent risk factor for RFS and OS in CRC patients. We used RCS to visualise the relationship between HCY levels and the risk of poor prognosis. We observed a continuous increase in the risk of RFS and OS mortality in CRC patients as HCY levels increased. We also found that patients in the high HCY level group (Q4) had a higher risk of adverse outcomes than those in the low HCY level group (Q1). We determined an optimal threshold value of 15.2 μmol/L for HCY levels for predicting the outcome of CRC patients. When the HCY level in CRC patients exceeded 15.2 μmol/L, the risks of adverse RFS and OS increased by 55.7% and 61.4%, respectively. We also discovered that patients with high HCY levels were more likely to be old, male, and have large tumours and high CEA levels. Additionally, these patients incurred higher hospitalisation costs and longer hospital stays, potentially indicating greater medical burden and expenses associated with high HCY levels. These findings highlighted HCY levels as a robust prognostic tool for predicting disease progression and survival in CRC patients.

Currently, the reasons for the elevation of HCY in CRC patients remain unclear and require further investigation. We hypothesize that elevated levels of HCY in the bloodstream may arise from heightened methylation and the production of *S*-adenosylmethionine upstream of HCY, or from the inhibition of the transsulfuration pathway (mediated by cystathionine β-synthase), or impaired remethylation of HCY back to methionine (involving methionine synthase) downstream of HCY.

Serum CEA level is the most commonly used tumour marker for disease diagnosis, therapeutic evaluation, and prediction of disease progression in CRC patients^24^. However, serum CEA levels in CRC patients are not specific, and a considerable proportion of patients have CEA levels within the normal range^25^. Additionally, CEA concentrations can be influenced by various factors such as smoking, liver disease, inflammation, and lung diseases, which may lead to false-positive or false-negative results^26^. Therefore, it is necessary to explore complementary serum markers to compensate for the limitations of CEA levels. Therefore, we investigated the prognostic value of HCY levels in different subgroups of CEA levels. We found that HCY levels effectively stratified patients according to prognosis, regardless of CEA negativity or positivity, with a more significant stratification observed in patients with high CEA levels. In addition, the combination of CEA and HCY can more effectively stratify the prognosis of CRC patients. Although TNM stage is the most widely used tool for evaluating CRC patients' prognosis, heterogeneity in patient prognosis still exists within the same pathological stage. In our study, we observed a significantly poorer prognosis in patients with elevated levels of HCY compared to those with lower levels, even within the same pathological stage. This observation suggests that measuring HCY levels could provide valuable additional information alongside pathological staging for CRC patients.

Survival nomograms integrate various demographic, serological, and pathological features to comprehensively assess patient prognosis. In this study, we employed LASSO regression analysis to select meaningful prognostic features and ultimately identified seven prognostic factors using Cox regression analysis. Based on these features, we constructed survival nomograms to predict the 1 to 5-year RFS and OS in CRC patients. These nomograms demonstrated favourable discrimination and calibration values. Compared with the traditional TNM staging system, these prognostic nomograms exhibited superior resolution and accuracy in predicting RFS and OS in CRC patients. These newly proposed prognostic models can assist clinicians to better quantify the risk of adverse prognosis in CRC patients, thereby providing guidance for individualised treatment strategies.

Our study had several limitations. Due to the retrospective nature of this study, we did not perform a sample size calculation, which is a limitation. However, we would like to highlight several strengths of our study, including a large sample size (1272 CRC patients), comprehensive collection of clinical and pathological data, and a long follow-up period (61.0 months). Furthermore, we explored multiple outcomes such as RFS and OS, and accounted for various confounding factors in the multivariable analysis. These considerations contribute to the robustness and reliability of our findings. Second, because of the nature of our study design, we could only observe the relationship between plasma HCY levels and CRC patient prognosis at a single time point and lacked continuous HCY level monitoring data. Finally, the prognostic nomogram proposed in our study can be widely applied in clinical practice only after external validation through large-scale, multicentre, prospective research.

## Conclusions

The findings of this study indicate that elevated levels of HCY are independent risk factors for adverse RFS and OS in CRC patients. In the prognostic assessment of CRC patients, HCY levels can provide valuable supplementary information regarding CEA levels and pathological staging. Prognostic models based on HCY levels are effective tools for the comprehensive evaluation of prognosis in CRC patients but require further external validation by additional cohorts.

### Supplementary Information

Supplementary Information.

## Data Availability

The datasets used and/or analysed during the current study are available from the corresponding author on reasonable request.
