# Supplementary material for: Elevated serum homocysteine levels associated with poor recurrence-free and overall survival in patients with colorectal cancer
Source: Sci Rep. 2024 May 2;14:10057. doi: 10.1038/s41598-024-60855-4 (PMC11066114; doi:10.1038/s41598-024-60855-4)
Supplement: Supplementary file 1 — Supplementary Information. [file 41598_2024_60855_MOESM1_ESM.docx]

**Figure S1.** The optimal threshold cutoff of homocysteine based on maximally selected rank statistics.

**
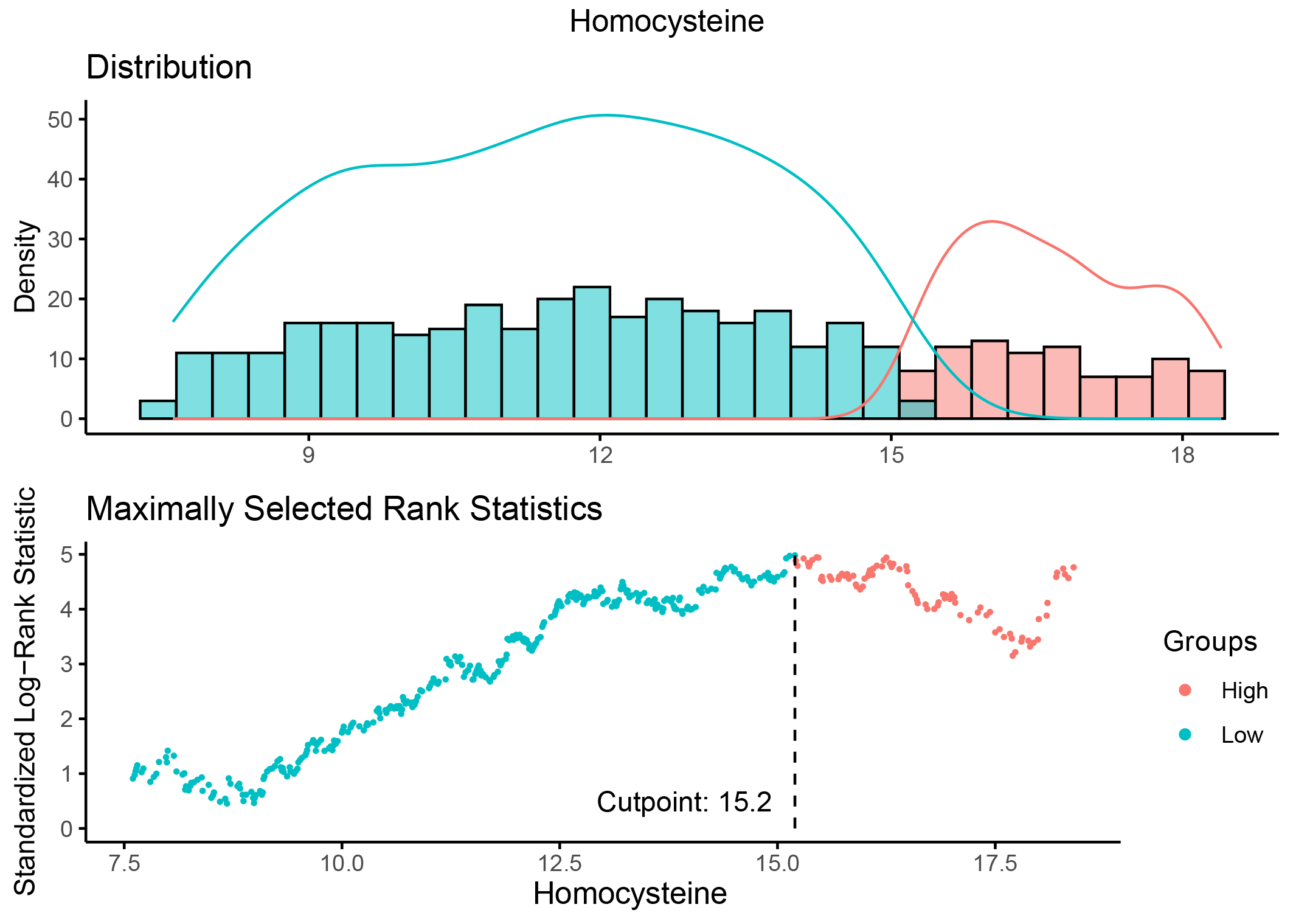
**

**Figure S2.** Median homocysteine of clinicopathological characteristics subgroup.

**
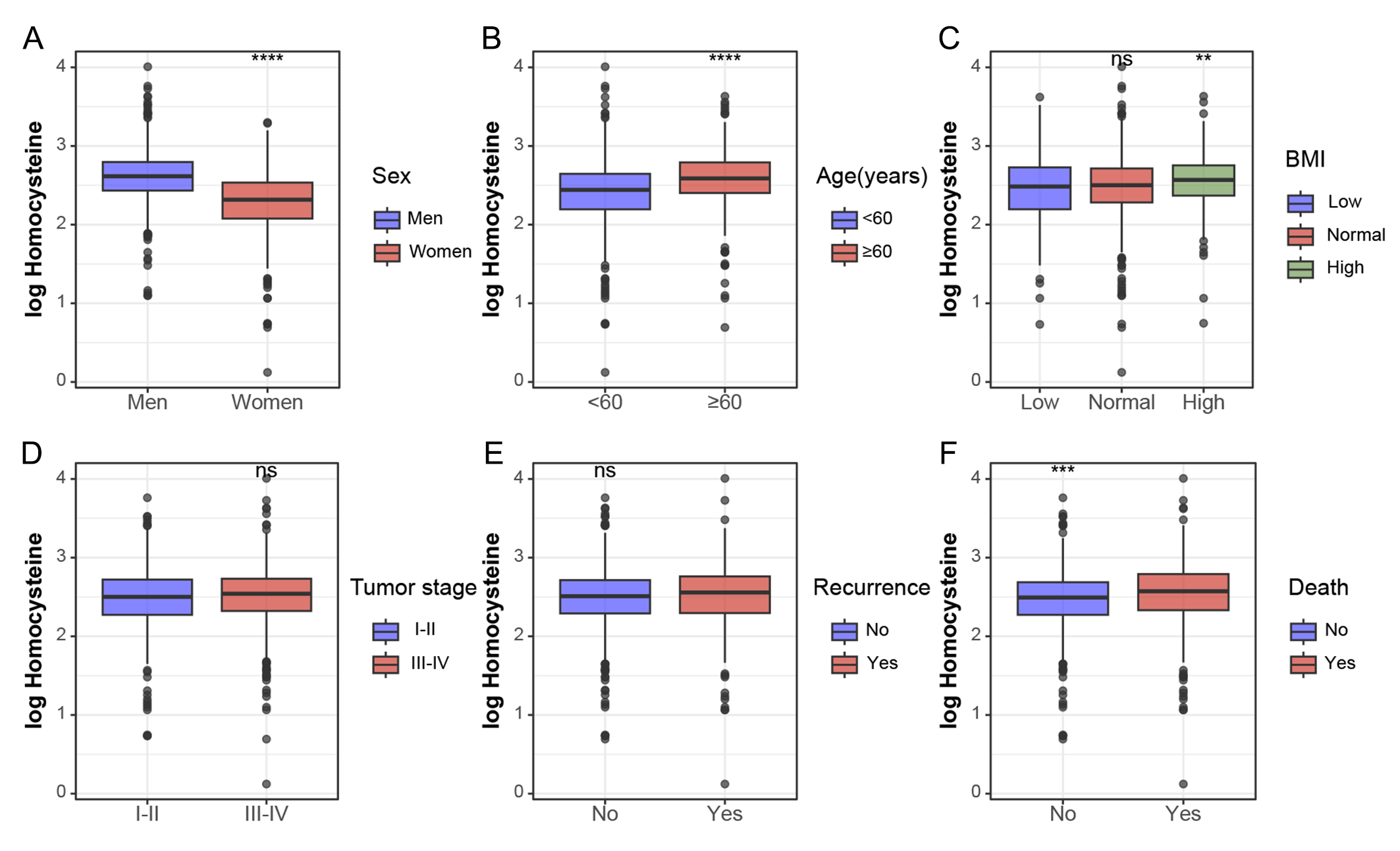
**

**Notes:** A, sex; B, age; C, BMI; D, tumor stage; E, recurrence; F, death.

**Figure S3.** Stratified Kaplan-Meier curve of homocysteine of patients with colon cancer.


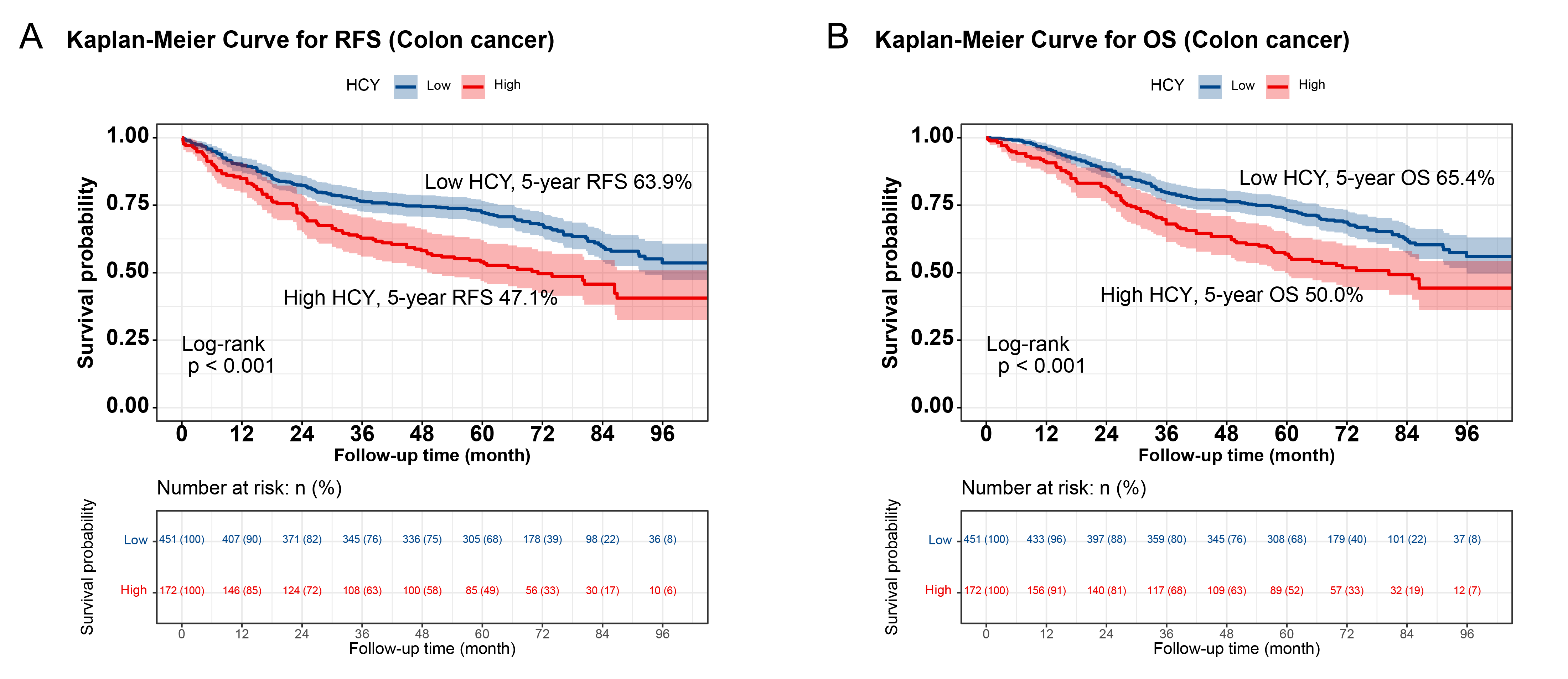


**Notes:** A, RFS; B, OS.

RFS, recurrence-free survival; OS, overall survival.

**Figure S4.** Stratified Kaplan-Meier curve of homocysteine of patients with rectal cancer.


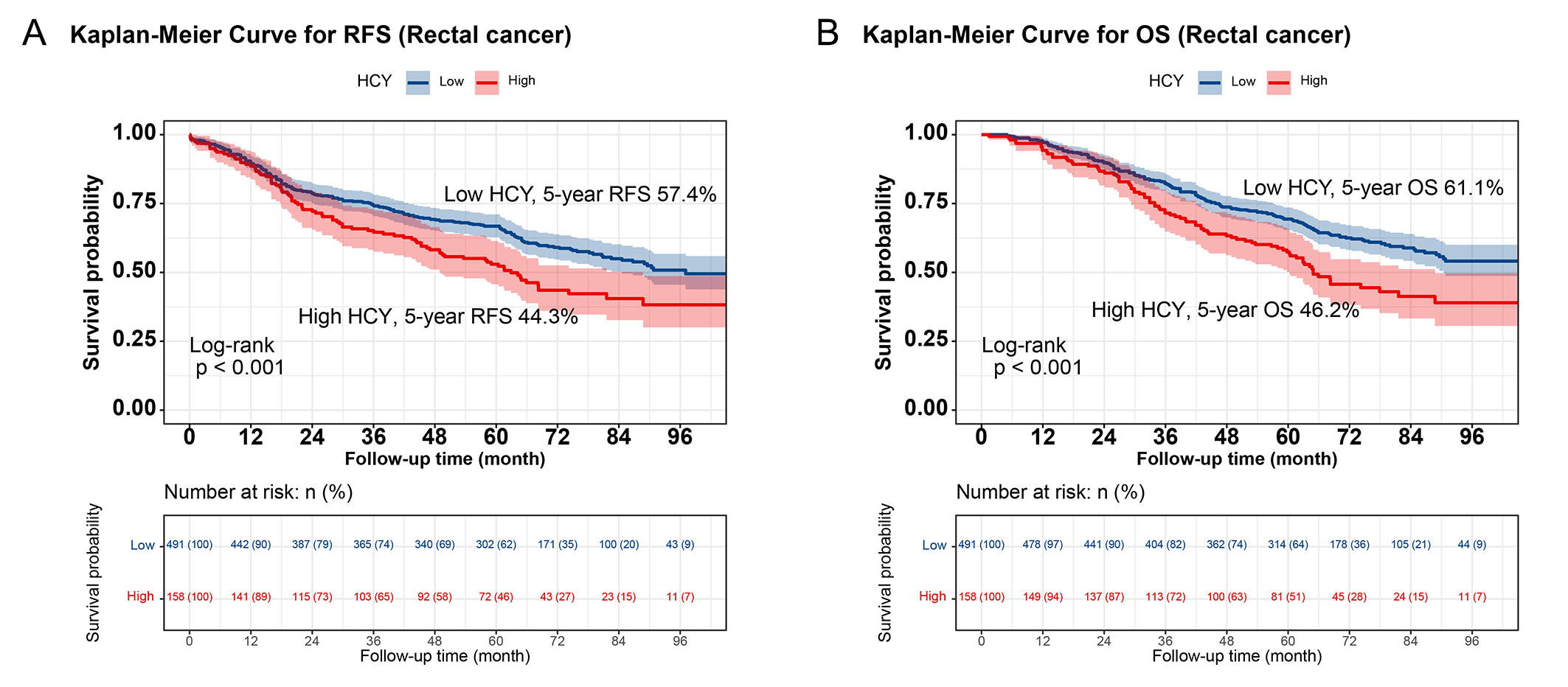


**Notes:** A, RFS; C, OS.

RFS, recurrence-free survival; OS, overall survival.

**Figure S5.** Stratified Kaplan-Meier curve of homocysteine in patients with normal CEA.


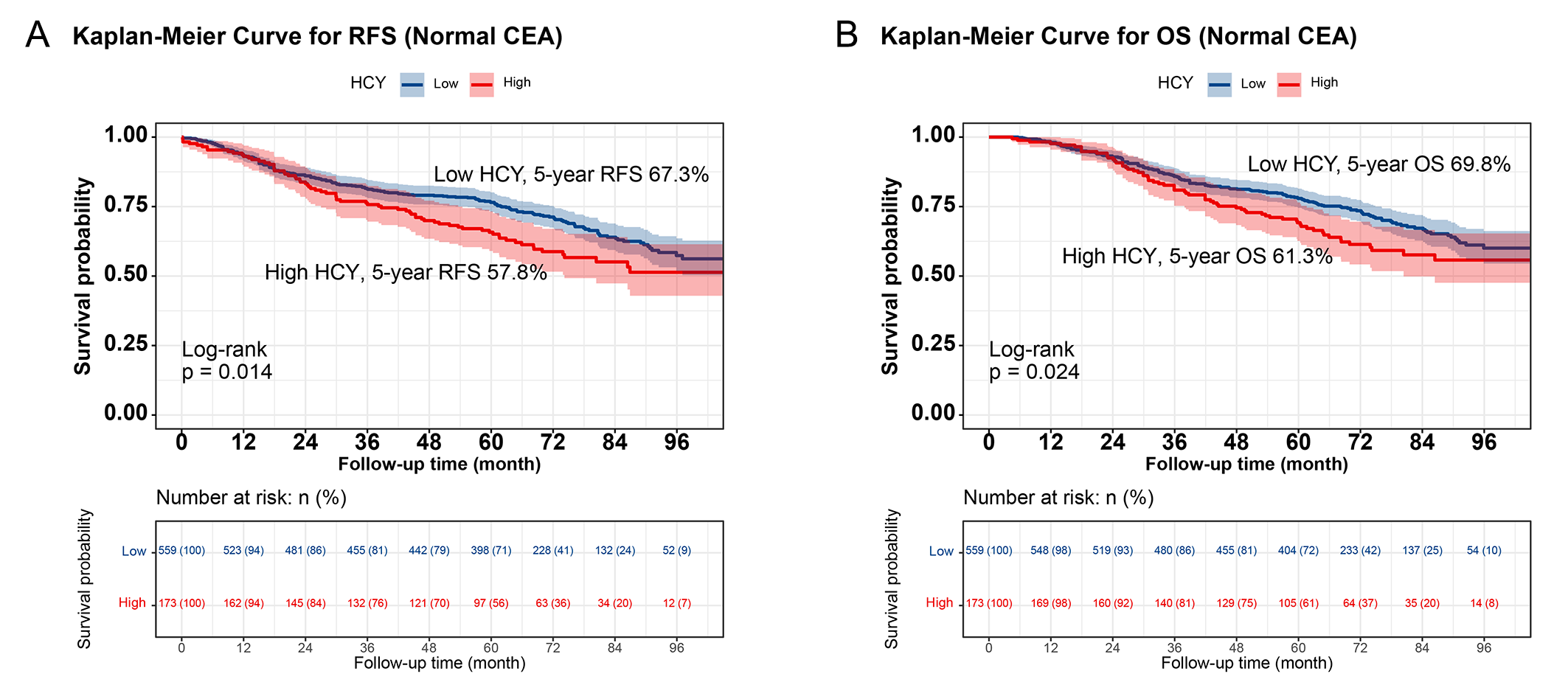


**Notes:** A, RFS; B, OS.

RFS, recurrence-free survival; OS, overall survival.

**Figure S6.** Stratified Kaplan-Meier curve of homocysteine in patients with high CEA.


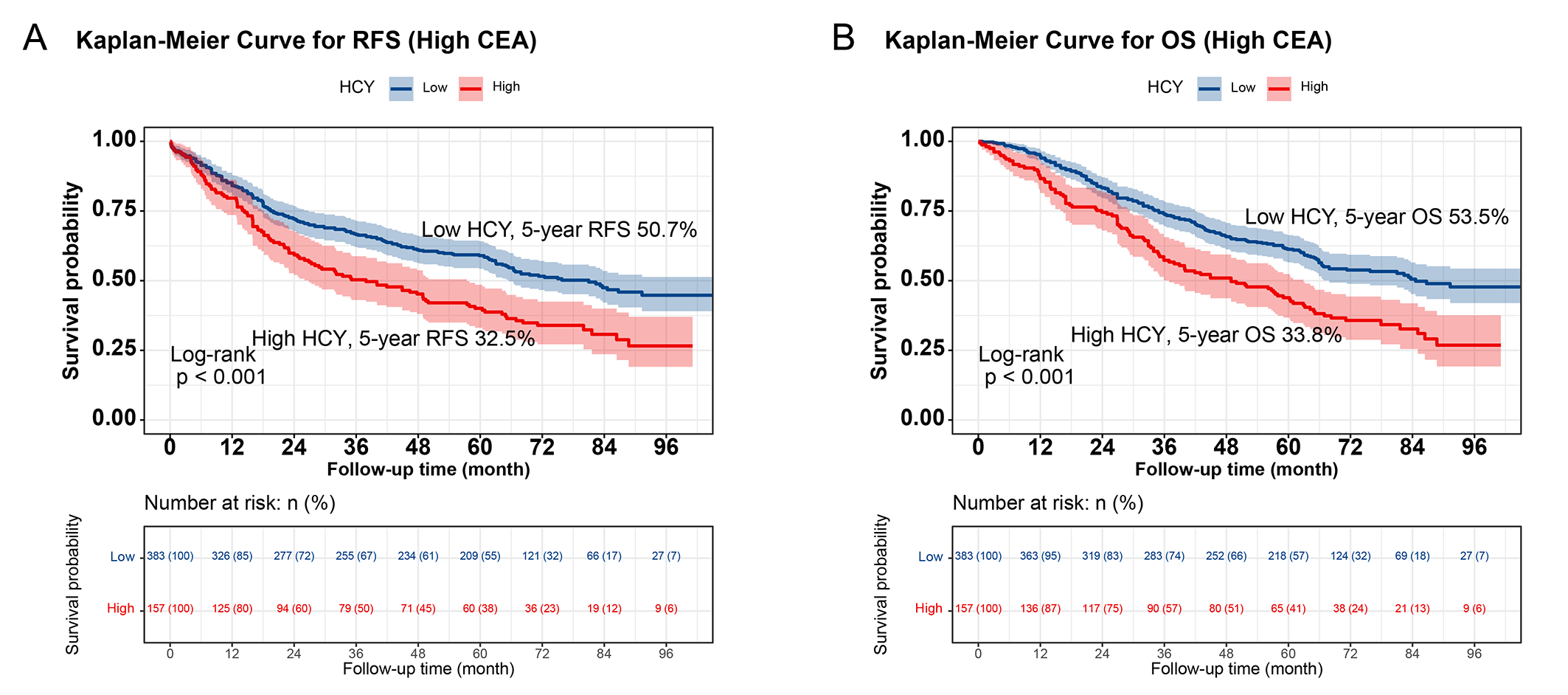


**Notes:** A, RFS; B, OS.

RFS, recurrence-free survival; OS, overall survival.

**Figure S7.** Kaplan-Meier curve of the combined effect of CEA and HCY

**
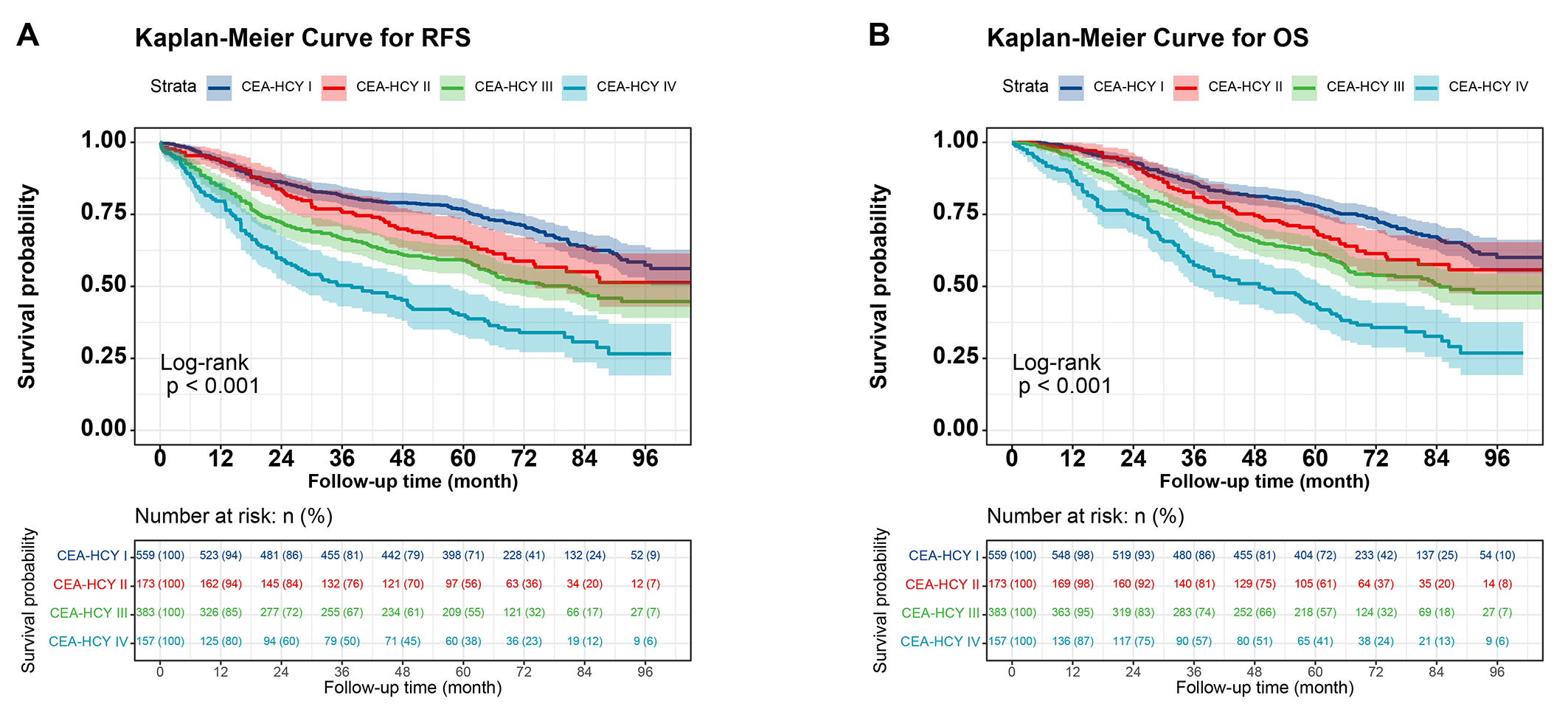
**

**Notes:** A, RFS; B, OS.

RFS, recurrence-free survival; OS, overall survival.

**Figure S8.** The association between homocysteine and hazard ratio of RFS in various subgroups.


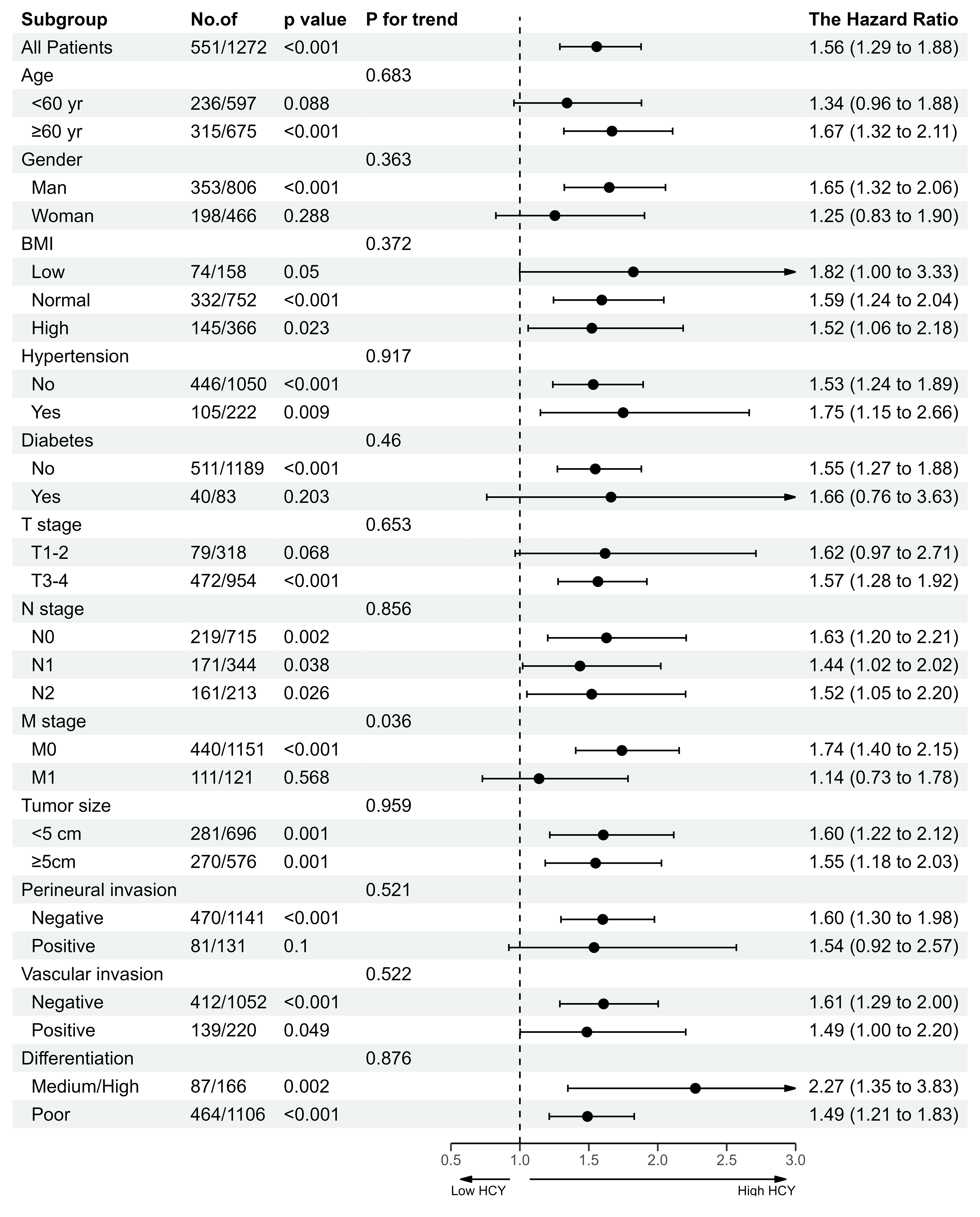


**Notes:** RFS, recurrence-free survival.

**Figure S9.** The association between homocysteine and hazard ratio of OS in various subgroups.


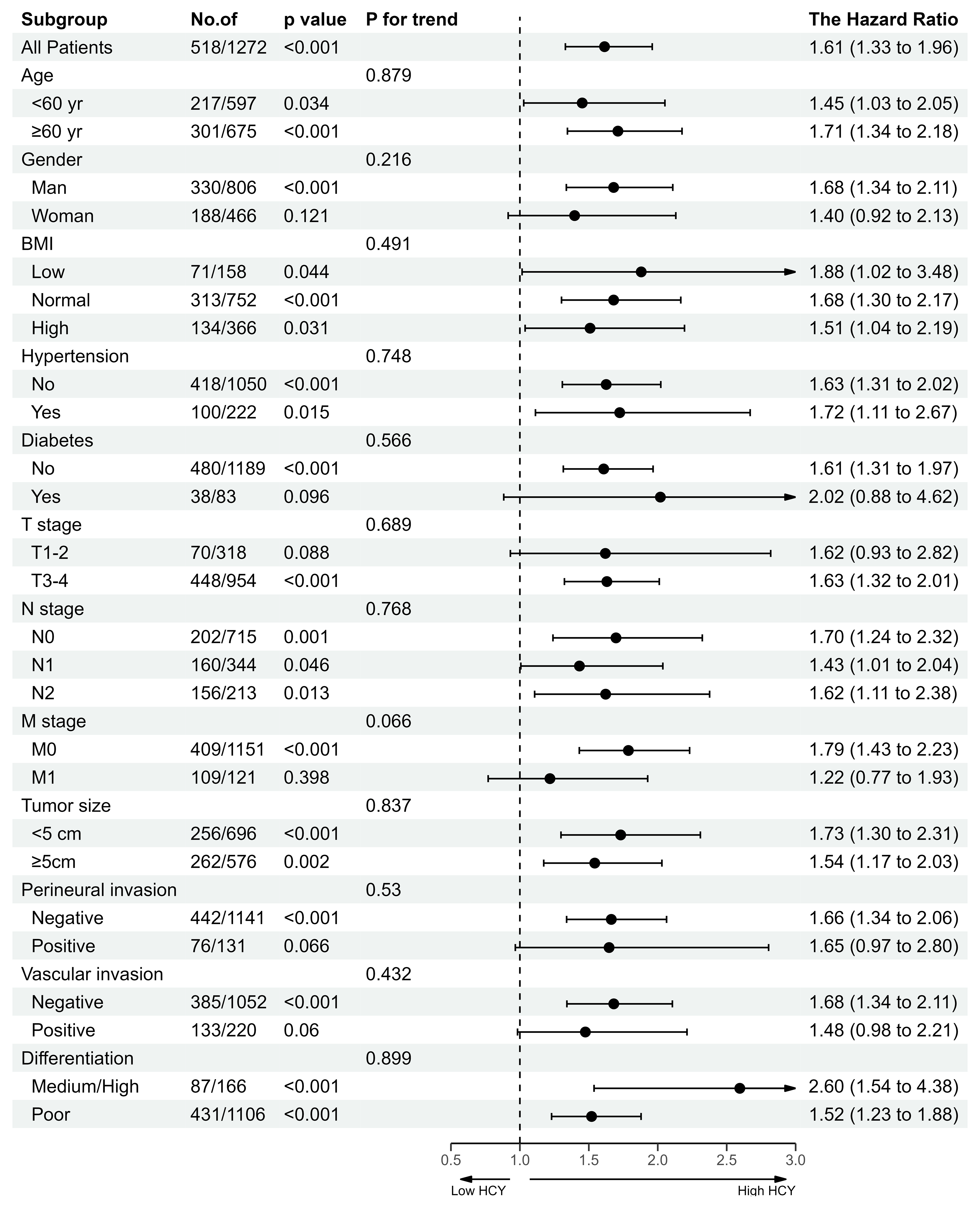


**Notes:** OS, overall survival.

**Figure S10.** Feature selection using least absolute shrinkage and selection operator (LASSO) logistic regression.

**
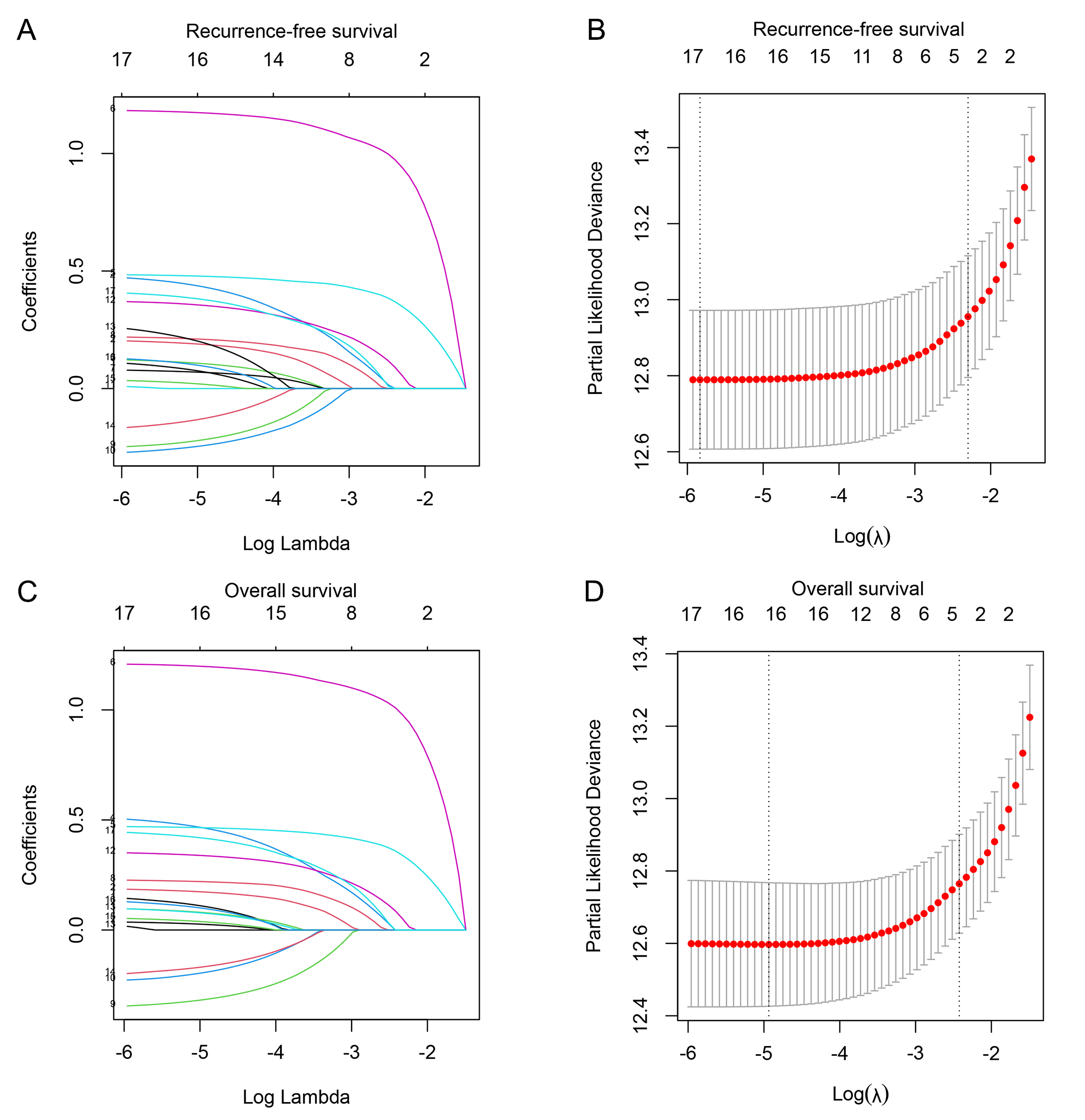
**

**Notes:** A-B, RFS; C-D, OS.

**Figure S11.** The 1-3 year ROC of RFS and OS Nomograms.

**
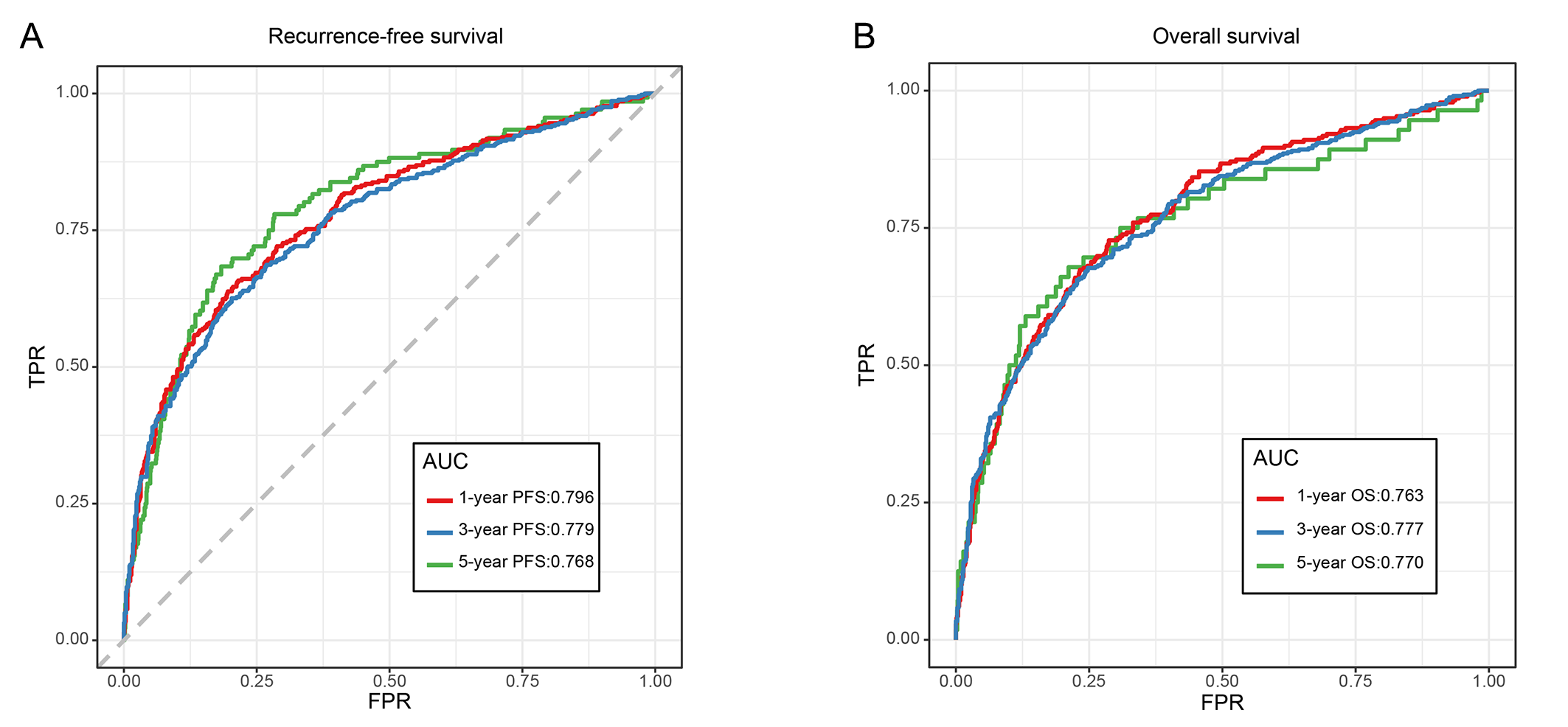
**

**Notes:** A, RFS; B, OS. ROC, receiver operating characteristic curve; RFS, recurrence-free survival; OS, overall survival. The red line represents 1-year ROC, the blue line represents 2-year ROC, and the green line represents 3-year ROC. The x-axis represents the false positive rate, and the y-axis represents the true positive rate. Any point on the curve represents the sensitivity and specificity corresponding to a specific positive threshold value of a screening test.

**Figure S12.** Calibration curve of the RFS and OS nomograms.

**
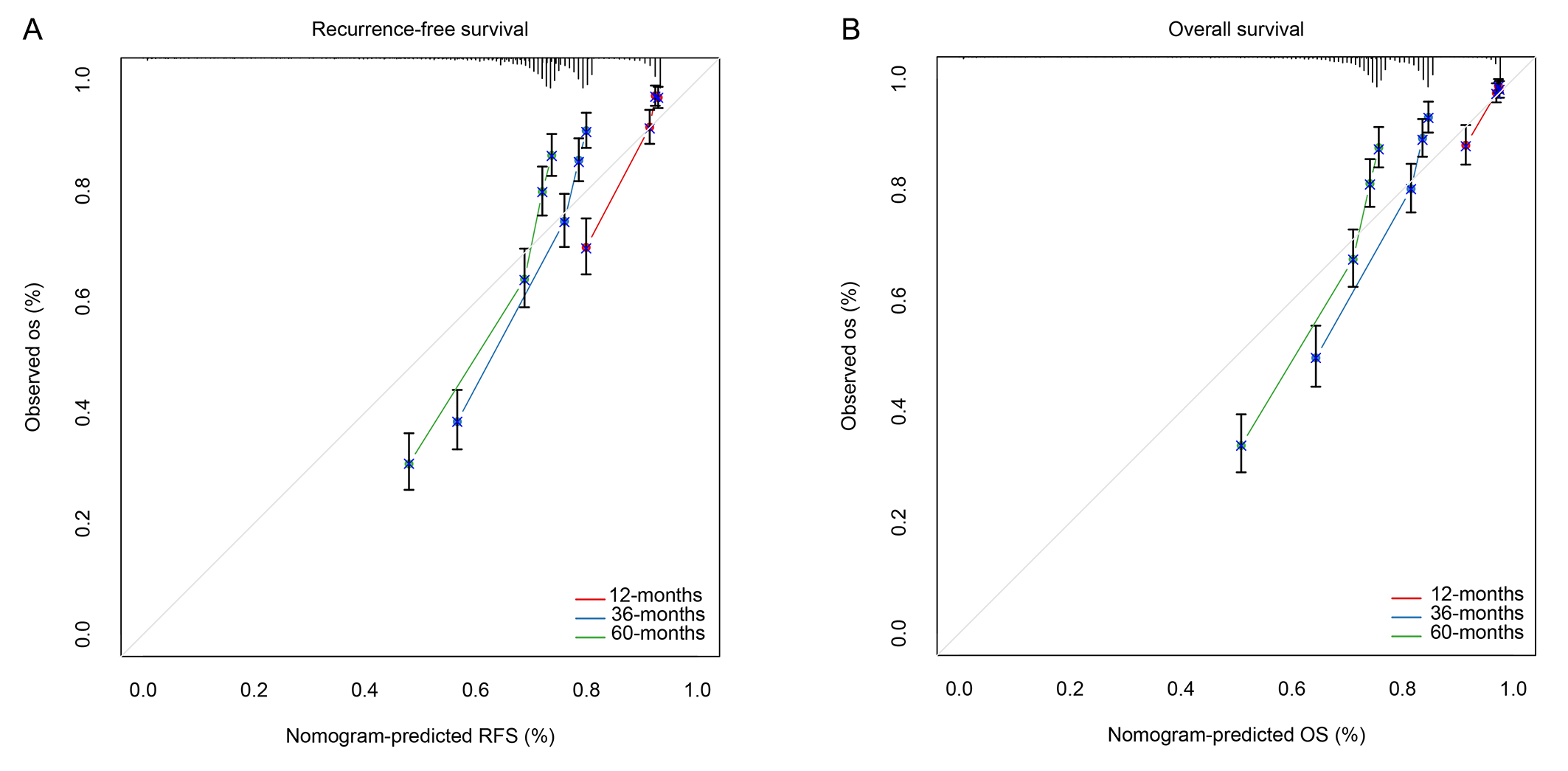
**

**Notes:** A, 1-3 year calibration curve of RFS; B, 1-3 year calibration curve of OS.

RFS, recurrence-free survival; OS, overall survival.

**Figure S13.** The DCA of RFS and OS nomograms.

**
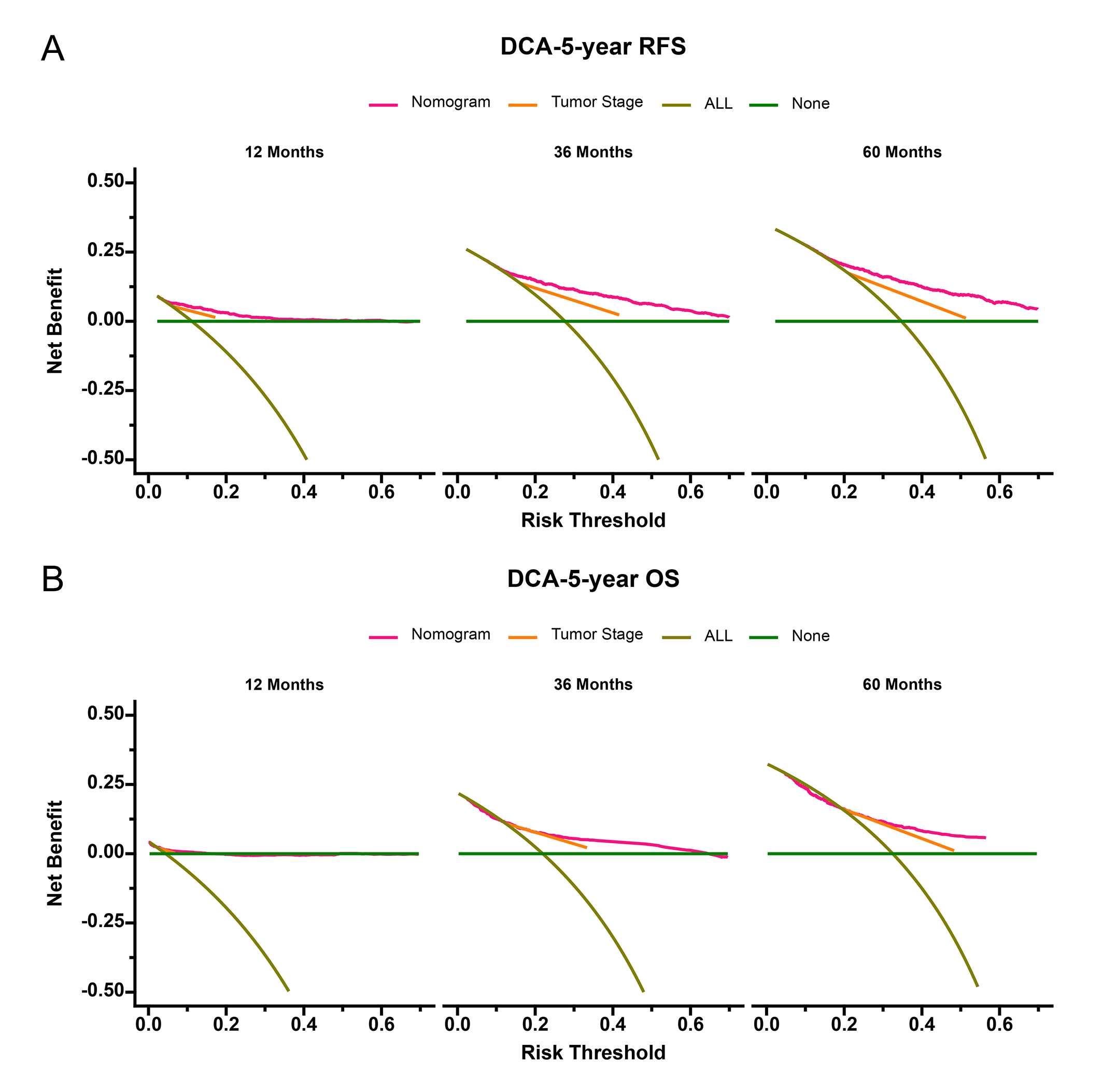
**

**Notes:** A, RFS; B, OS.

DCA, decision curve analysis; RFS, recurrence-free survival; OS, overall survival.

**Figure S14.** Kaplan-Meier curve of low and high score of nomograms in patients with colorectal cancer.

**
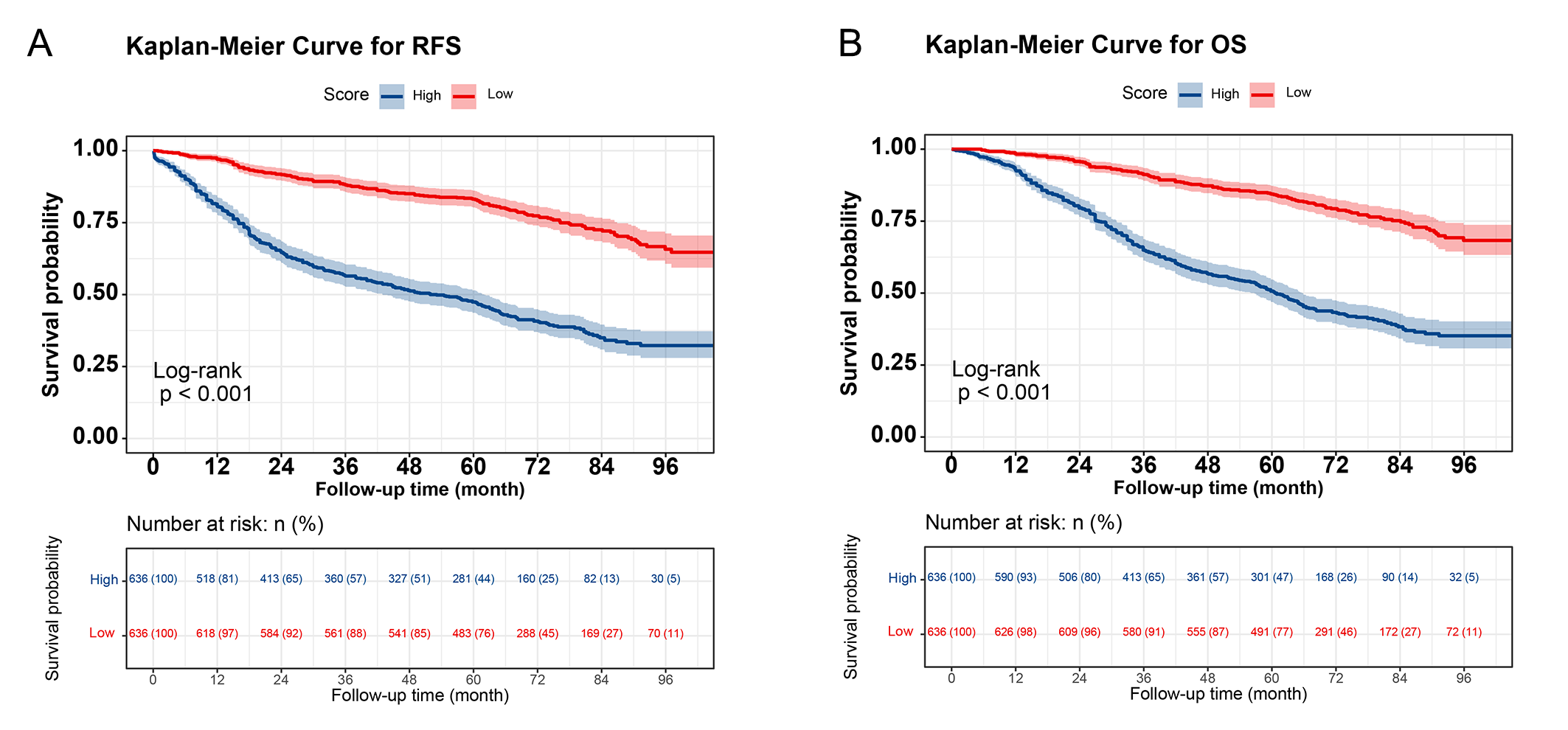
**

**Notes:** A, RFS nomogram; B, OS nomogram.

RFS, recurrence-free survival; OS, overall survival.

**Table S1** Clinicopathological characteristics of patients with colorectal cancer.

| Clinicopathological characteristics | Overall | Low HCY | High HCY | p |
| --- | --- | --- | --- | --- |
|  | n=1272 | n=942 | n=330 |  |
| Sex (Man) | 806 (63.4) | 528 (56.1) | 278 (84.2) | <0.001 |
| Age (mean (SD)) | 59.20 (12.65) | 57.68 (12.21) | 63.56 (12.88) | <0.001 |
| BMI (median [IQR]) | 22.07 (20.00, 24.44） | 22.03 (19.97, 24.22） | 22.36 (20.20, 24.76） | 0.165 |
| Hypertension (Yes) | 222 (17.5) | 144 (15.3) | 78 (23.6) | 0.001 |
| Diabetes (Yes) | 83 (6.5) | 65 (6.9) | 18 (5.5) | 0.432 |
| T stage (T3-4) | 954 (75.0) | 714 (75.8) | 240 (72.7) | 0.301 |
| N stage |  |  |  | 0.542 |
| N0 | 715 (56.2) | 530 (56.3) | 185 (56.1) |  |
| N1 | 344 (27.0) | 260 (27.6) | 84 (25.5) |  |
| N2 | 213 (16.7) | 152 (16.1) | 61 (18.5) |  |
| M stage | 121 (9.5) | 83 (8.8) | 38 (11.5) | 0.183 |
| TNM stage (III-IV) | 593 (46.6) | 437 (46.4) | 156 (47.3) | 0.832 |
| Perineural invasion (Yes) | 131 (10.3) | 90 (9.6) | 41 (12.4) | 0.170 |
| Vascular invasion (Yes) | 220 (17.3) | 160 (17.0) | 60 (18.2) | 0.682 |
| Differentiation (Poor) | 166 (13.1) | 126 (13.4) | 40 (12.1) | 0.626 |
| Location (Rectal cancer) | 649 (51.0) | 491 (52.1) | 158 (47.9) | 0.206 |
| Tumor size (median [IQR]) | 4.78 (2.09) | 4.72 (2.07) | 4.97 (2.12) | 0.059 |
| CEA (median [IQR]) | 3.94 [2.11, 11.38] | 3.70 [1.98, 10.62] | 4.63 [2.57, 13.71] | 0.004 |
| Radiotherapy (%) | 110 (8.6) | 87 (9.2) | 23 (7.0) | 0.252 |
| Chemotherapy (%) | 565 (44.4) | 427 (45.3) | 138 (41.8) | 0.298 |
| Death (Yes) | 518 (40.7) | 347 (36.8) | 171 (51.8) | <0.001 |
| Recurrence (Yes) | 349 (27.4) | 243 (25.8) | 106 (32.1) | 0.032 |
| Length of stay (median [IQR]) | 17.00 (11.00, 21.00） | 16.00 (10.25, 20.00） | 18.00 (14.00, 23.00） | <0.001 |
| Hospitalization cost (median [IQR]) | 49886.36 (44827.78, 56384.45） | 49360.30 (44507.49, 55586.76） | 51593.22 (46091.48, 60090.52） | <0.001 |

Table Note: CRC, colorectal cancer; BMI, body mass index; HCY, homocysteine.

**Table S2.** The combined effect of CEA and homocysteine.

| IBI | Model a | p value | Model b | p value | Model c | p value |
| --- | --- | --- | --- | --- | --- | --- |
| CEA-HCY I | ref |  | ref |  | ref |  |
| CEA-HCY II | 1.388 (1.058,1.821) | 0.018 | 1.514 (1.144,2.004) | 0.004 | 1.596 (1.203,2.118) | 0.001 |
| CEA-HCY III | 1.791 (1.461,2.194) | <0.001 | 1.431 (1.163,1.761) | 0.001 | 1.496 (1.212,1.846) | <0.001 |
| CEA-HCY IV | 2.950 (2.321,3.750) | <0.001 | 2.074 (1.610,2.673) | <0.001 | 2.203 (1.701,2.852) | <0.001 |
| p for trend |  | <0.001 |  | <0.001 |  | <0.001 |

Notes:

Model a: No adjusted.

Model b: Adjusted for age, sex, BMI, T stage, N stage, metastasis,.

Model c: Adjusted for sex, age, BMI, hypertension, diabetes, T stage, N stage, M stage, tumor location, tumor size, perineural invasion, vascular invasion, differentiation, radiotherapy, chemotherapy, hypertension, diabetes, family history.

**Table S3.** Univariate and multivariate Cox regression analysis of clinicopathological characteristics associated with recurrence-free survival in CRC patients.

| Characteristic | Recurrence-free survival | | | |
| --- | --- | --- | --- | --- |
|  | Univariate analysis | | Multivariate analysis | |
|  | HR (95%CI) | P value | HR (95%CI) | P value |
| Sex | 1.034 (0.869-1.230) | 0.708 |  |  |
| Age | 1.249 (1.055,1.478) | 0.010 | 1.268 (1.065,1.510) | 0.008 |
| BMI |  | 0.143 |  |  |
| Low | Ref. |  |  |  |
| Normal | 1.210 (0.940-1.556) | 0.138 |  |  |
| High | 1.328 (1.002-1.760) | 0.048 |  |  |
| T stage | 2.419 (1.905-3.070) | <0.001 | 1.531 (1.186-1.976) | 0.001 |
| N stage |  | <0.001 |  | <0.001 |
| N0 | Ref. |  | Ref. |  |
| N1 | 1.870 (1.531-2.284) | <0.001 | 1.488 (1.208-1.833) | <0.001 |
| N2 | 4.111 (3.349-5.046) | <0.001 | 2.723 (2.163-3.428) | <0.001 |
| M stage | 5.282 (4.269-6.536) | <0.001 | 3.140 (2.507-3.934) | <0.001 |
| Perineural invasion (Yes) | 1.951 (1.584-2.403) | <0.001 | 1.113 (0.854-2.403) | 0.429 |
| Vascular invasion (Yes) | 2.060 (1.698-2.498) | <0.001 | 1.238 (0.989-1.549) | 0.063 |
| Differentiation (Poor) | 1.492 (1.186-1.876) | 0.001 | 1.268 (1.001-1.608) | 0.049 |
| Location (Rectal cancer) | 1.157 (0.978-1.368) | 0.088 |  |  |
| Tumor size (median [IQR]) | 1.205 (1.019-1.424) | 0.029 | 0.954 (0.803-1.134) | 0.597 |
| CEA (≥5ng/ml) | 1.916 (1.620-2.266) | <0.001 | 1.441 (1.206-2.722) | <0.001 |
| Radiotherapy (Yes) | 1.264 (0.954-1.674) | 0.102 |  |  |
| Chemotherapy (Yes) | 1.099 (0.929-1.300) | 0.271 |  |  |
| Hypertension (Yes) | 1.151 (0.931-1.424) | 0.194 |  |  |
| Diabetes (Yes) | 1.150 (0.834-1.587) | 0.393 |  |  |
| HCY (High) | 1.566 (1.310-1.872) | <0.001 | 1.470 (1.225-1.764) | <0.001 |

Table Note: CRC, colorectal cancer; BMI, body mass index.

**Table S4** Univariate and multivariate Cox regression analysis of clinicopathological characteristics associated with overall survival in CRC patients.

| Characteristic | Overall survival | | | |
| --- | --- | --- | --- | --- |
|  | Univariate analysis | | Multivariate analysis | |
|  | HR (95%CI) | P value | HR (95%CI) | P value |
| Sex | 1.012 (0.846-1.211) | 0.896 |  |  |
| Age | 1.304 (1.095,1.553) | 0.003 | 1.288 (1.075,1.542) | 0.006 |
| BMI |  | 0.291 |  |  |
| Low | Ref. |  |  |  |
| Normal | 1.154 (0.892-1.493) | 0.276 |  |  |
| High | 1.260 (0.944-1.682) | 0.116 |  |  |
| T stage | 2.549 (1.981-3.279) | <0.001 | 1.575 (1.203-2.062) | 0.001 |
| N stage | Ref. | <0.001 |  |  |
| N0 |  |  |  |  |
| N1 | 1.863 (1.514-2.293) | <0.001 | 1.482 (1.194-1.840) | <0.001 |
| N2 | 4.135 (3.350-5.104) | <0.001 | 2.600 (2.051-3.295) | <0.001 |
| M stage | 5.446 (4.388-6.758) | <0.001 | 3.261 (2.595-4.097) | <0.001 |
| Perineural invasion (Yes) | 1.793 (1.405-2.288) | <0.001 | 1.070 (0.813-1.408) | 0.629 |
| Vascular invasion (Yes) | 2.067 (1.696-2.519) | <0.001 | 1.256 (0.997-1.582) | 0.053 |
| Differentiation (Poor) | 1.625 (1.290-2.047) | <0.001 | 1.412 (1.111-1.794) | 0.005 |
| Location (Rectal cancer) | 0.912 (0.767-1.084) | 0.296 |  |  |
| Tumor size (median [IQR]) | 1.322 (1.113-1.571) | 0.001 | 1.063 (0.890-1.270) | 0.503 |
| CEA (≥5ng/ml) | 1.956 (1.645-2.326) | <0.001 | 1.435 (1.194-1.724) | <0.001 |
| Radiotherapy (Yes) | 1.214 (0.912-1.876) | 0.251 |  |  |
| Chemotherapy (Yes) | 1.015 (0.853-1.208) | 0.864 |  |  |
| Hypertension (Yes) | 1.178 (0.947-1.466) | 0.141 |  |  |
| Diabetes (Yes) | 1.174 (0.844-1.633) | 0.342 |  |  |
| HCY (High) | 1.610 (1.340-1.934) | <0.001 | 1.522 (1.262-1.835) | <0.001 |

Table Note: CRC, colorectal cancer; BMI, body mass index.
